# Expression Dynamics of Neurotransmitter System Genes in Early Sea Urchin Embryos: Insights from a Four-Species Comparative Transcriptome Analysis

**DOI:** 10.3390/biology14091262

**Published:** 2025-09-12

**Authors:** Yuri B. Shmukler, Nina M. Alyoshina, Yulia O. Nikishina, Denis A. Nikishin

**Affiliations:** Koltzov Institute of Developmental Biology RAS, Vavilov Street, 26, Moscow 119334, Russia; n.alyoshina@idbras.ru (N.M.A.); y.nikishina@idbras.ru (Y.O.N.)

**Keywords:** sea urchin, early development, transcriptome, neurotransmitter systems, comparative transcriptomics, serotonin, dopamine, acetylcholine, glutamate, interspecies variability

## Abstract

Transmitters such as serotonin, dopamine, noradrenaline, and acetylcholine act as regulators or triggers of numerous processes in the early embryo, including in sea urchins. However, the identity of these mechanisms relative to mature nervous systems remains controversial. The aim of this study was to comprehensively characterize the transcriptomic basis of these as well as glutamatergic, GABAergic and histaminergic systems by comparing publicly available RNA-Seq data across four sea urchin species *(Mesocentrotus franciscanus*, *Lytechinus variegatus*, *Paracentrotus lividus*, *Strongylocentrotus purpuratus*) during early development (egg to early gastrula). Transcript abundance was normalized using the geometric mean of housekeeping genes (GHG) to facilitate comparative analysis and to use the universal significance threshold. We detected mRNA transcripts encoding numerous components (enzymes, receptors, transporters) for all seven transmitter systems from the earliest stages, suggesting a complex signaling potential prior to neurogenesis. The expression of multiple mRNAs of receptors for the same transmitter indirectly supports our earlier notion of the possibility of simultaneous regulation of different processes by this transmitter even in the single-cell embryo. Notably, transcripts for key synthesis enzymes (TPH, DBH) were often low, indicating limited de novo synthesis, while transcripts for degradation enzymes (MAO, AChE) were abundant. Consistent expression across species was observed for specific receptors such as HTR6, D1-like dopamine, β-adrenergic receptors and the α7 subunit of nicotinic AChR. However, the expression profiles of many components, particularly glutamatergic receptors and metabolic enzymes, showed considerable interspecies variability. These findings indicate that multiple transmitter systems are transcriptionally represented early in development, suggesting substantial molecular overlap with mature systems, while the diversity between species points to possible evolutionary plasticity. This comparative transcriptomic dataset provides a basis for targeted functional studies of the role and interactions of these pre-nervous transmitter pathways in orchestrating embryogenesis.

## Simple Summary

This research explores how key communication systems, similar to those in our brains, are present in the very early stages of sea urchin development, even before they have a nervous system. Scientists have long known that substances like serotonin and dopamine play roles in early development. This study examined four different sea urchins, comparing information about their genes to understand how these communication systems are set up. The study found that the instructions for making these systems are present from the very beginning, suggesting they are important for guiding early development. There were also differences between the sea urchin species, hinting at how these systems might have changed evolutionary. This information can help us understand how these communication systems work in early development and could have implications for understanding development in other animals, including humans. This is valuable because it shows that these systems are important not just for the brain, but also for fundamental processes of life.

## 1. Introduction

Pioneering research conducted from the 1950s to the early 1960s demonstrated both the presence and functional activity of several classical transmitters during early development (for a comprehensive overview, see [1]). Serotonin (5-HT) was among the first neurotransmitters identified in sea urchin embryos. Its presence was initially detected through fluorometry and immunohistochemistry [2], and later confirmed in unfertilized eggs using high-performance liquid chromatography (HPLC) [3].

Since this foundational work, the early sea urchin embryo has become a valuable model system for investigating the role of neurotransmitter-like substances in early development. This is largely due to its relatively large, optically transparent, and rapidly and synchronously dividing embryos, whose cleavage divisions are sensitive to neuropharmaceutical agents [4].

Further studies have revealed that physiological concentrations of transmitters, particularly serotonin, interact with their receptors to influence fundamental developmental processes. These processes include cleavage divisions, blastomere interaction, and morphogenesis (for a review, see [1,4,5,6]). However, despite decades of embryopharmacological studies, the molecular basis of these effects remains unclear.

Dopamine represents another key neurotransmitter identified in sea urchin embryos [3,7,8,9]. Studies have shown that dopamine receptor antagonists exhibit embryostatic effects, and that dopamine is involved in regulating embryonic motility [4,10,11].

In contrast to the extensive attention given to serotonin and dopamine, the adrenergic system in the early sea urchin embryo has received comparatively little study. Nevertheless, embryophysiological experiments have documented embryostatic effects induced by various adrenoreceptor antagonists, notably β-adrenolytics [4]. Interestingly, unlike serotonin receptor antagonists, β-adrenergic receptor antagonists block cleavage divisions by increasing cytocortex rigidity. This suggests that the natural function of adrenergic transmitters is to reduce cytocortex rigidity, whereas serotonin appears to increase it [12].

The cholinergic system is considered the first transmitter system identified in early sea urchin embryos [13,14]. Subsequent research demonstrated the embryostatic effects of cholinolytics on cleaving embryos [15,16]. Detailed investigations also revealed sophisticated cholinergic mechanisms in sea urchin fertilization [17,18,19].

While significant progress has been made in understanding the role of serotonergic, dopaminergic, adrenergic, and cholinergic systems, other potential transmitter systems active in the early sea urchin embryo remain poorly characterized. These systems clearly warrant further detailed investigation.

Analyzing neurotransmitter pathways in early development, from the first cleavages to the early gastrula stage, is of particular interest. This contrasts with many pharmacological experimental studies that primarily focus on larvae, where neural cells are already present. A central question in this field is whether early embryonic transmitter mechanisms are functionally identical to their counterparts in the mature nervous system, or if they represent distinct, developmentally specialized molecular structures [20]. Early pharmacological studies, which focused on ligand binding properties and effective concentration ranges, indicated potentially significant differences in embryonic receptor mechanisms compared to adult forms. Another reason for this caution was the probable intracellular localization of embryonic transmitter receptors [4,21], which deviates from classical neurotransmitter receptors.

Modern molecular biology techniques, particularly comprehensive transcriptome analysis, offer a powerful approach to directly address this question. They allow for the characterization of expression profiles of genes encoding components of transmitter mechanisms in early embryonic cells. By comparing transcriptomic datasets across multiple sea urchin species, we can identify evolutionarily conserved components of these pre-nervous transmitter systems. This, in turn, provides insights into the proteins likely responsible for the observed physiological functions. Furthermore, a comparative analysis of transcriptomes from four different sea urchin species enhances the robustness of our conclusions and facilitates the identification of species-specific adaptations or variations. The present study specifically focuses on analyzing transcriptome data related to serotonergic, dopaminergic, adrenergic, cholinergic, glutamatergic, GABAergic, and histaminergic mechanisms within this comparative framework.

Recent comparative transcriptomic studies in invertebrates have further highlighted the evolutionary conservation and diversification of neurotransmitter systems [22], underscoring the broader significance of our findings. These transcriptomic patterns open new avenues for understanding how neurotransmitter signaling may coordinate early developmental events independently of their canonical neuronal functions. They also inform future studies that integrate these findings with classical approaches in experimental embryology. These results have far-reaching implications for evolutionary developmental biology, neurobiology, and biomedical research, potentially informing investigations into the role of neurotransmitters in cellular communication beyond synaptic connections.

## 2. Materials and Methods

### 2.1. Specimen Selection and Transcriptome Data

The genome of *Strongylocentrotus purpuratus* was the first to be sequenced among sea urchins [23] and represents a fundamental reference for subsequent genomic and transcriptomic studies in echinoderms. In the present work, we performed a comparative analysis with publicly available transcriptome data of four sea urchin species: *Mesocentrotus franciscanus* [24], *Lytechinus variegatus* [25], *Paracentrotus lividus* [26], and *Strongylocentrotus purpuratus* [27]. The assembled *M. franciscanus* transcriptome is available in the NCBI Transcriptome Shotgun Assembly (TSA) database under accession GHJZ00000000. Additional data files, including the transcriptome annotation, can be accessed at [24]. Data for *P. lividus* were extracted from supplemental files associated with the publication by [26]. Data for the *L. variegatus* transcriptome were obtained from the open-access database https://lvedge.bu.edu/cgi-bin/lvedge/main.py (accessed on 24 August 2024). Data for the *S. purpuratus* transcriptome were sourced from the open-access database https://download.xenbase.org/echinobase/Legacy/ (accessed on 17 May 2025). Due to the nature of the data (RNA-Seq) and the developmental stages recorded, these datasets were considered sufficiently compatible to allow a comparative analysis of gene expression dynamics in the four species.

To assess expression dynamics during early development, we analyzed transcriptome data from key developmental milestones common to all four species datasets, where available. These stages typically included: early cleavage (approximately 1–16 cells), late cleavage (approximately 60 cells), early blastula, late blastula (encompassing hatching and mesenchyme-blastula stages), and early gastrula. These stages were selected because they collectively encompass the critical maternal-to-zygotic transition (MZT) in sea urchins. During the MZT, developmental control shifts from maternally deposited molecules to products of the newly activated zygotic genome (Table 1 summarizes specific time points for each species). In *S. purpuratus*, a minor wave of zygotic transcription occurs during early cleavage divisions, while the major wave of zygotic transcription takes place around the blastula stage [28]. We considered the 16-cell stage as part of the ‘early cleavage’ period. Similar to *S. purpuratus*, *M. franciscanus* also exhibits a major wave of zygotic transcription at the blastula stage [24]. The main wave of the MZT occurs at the blastula stage in most sea urchin species [29].

### 2.2. Normalization and Thresholding of Transcriptome Data

To ensure comparability across the four different transcriptome datasets, which originally reported transcript abundance in various units (e.g., FPKM, TPM), we implemented a normalization procedure for gene expression levels within each species dataset. Normalization was performed relative to the geometric mean of the expression levels of three commonly used housekeeping genes (HKGs): Glyceraldehyde-3-phosphate dehydrogenase (*GAPDH*), ornithine decarboxylase (*ODC*), and hypoxanthine-guanine phosphoribosyltransferase (*HPRT*). These specific HKGs were selected because they were represented and annotated in all four examined transcriptome datasets (Table 2 presents the calculated geometric mean values used for normalization at each stage). Specifically, the mRNA abundance for each gene of interest at each developmental stage was expressed as a fraction of the geometric mean values of these three HKGs from the same stage. Using the geometric mean of multiple stable housekeeping genes is recognized as an accurate normalization method for gene expression analysis [30]. This normalization strategy accounted for potential variations in sequencing depth, library size, and overall transcriptional activity among different samples and species.

Following the precedent established by [27] for analyzing *S. purpuratus* transcriptomes, we applied an expression threshold to filter out genes with very low abundance. Such genes are less likely to be biologically significant and may represent transcriptional noise. A threshold of approximately 300 transcripts per embryo (roughly equivalent to FPKM ~5 in the original *S. purpuratus* data) was chosen as the lower limit for a transcript’s expression to be considered potentially functionally relevant. This absolute threshold corresponds to a normalized value of approximately 0.003 GHG using our normalization procedure. Transcripts with normalized expression values below this threshold were generally excluded from detailed analysis or considered negligible. All digital data for the serotonergic system transcriptomes are summarized in Supplementary Tables S1–S7.

## 3. Results

### 3.1. Serotonergic System

**Figure 1:**
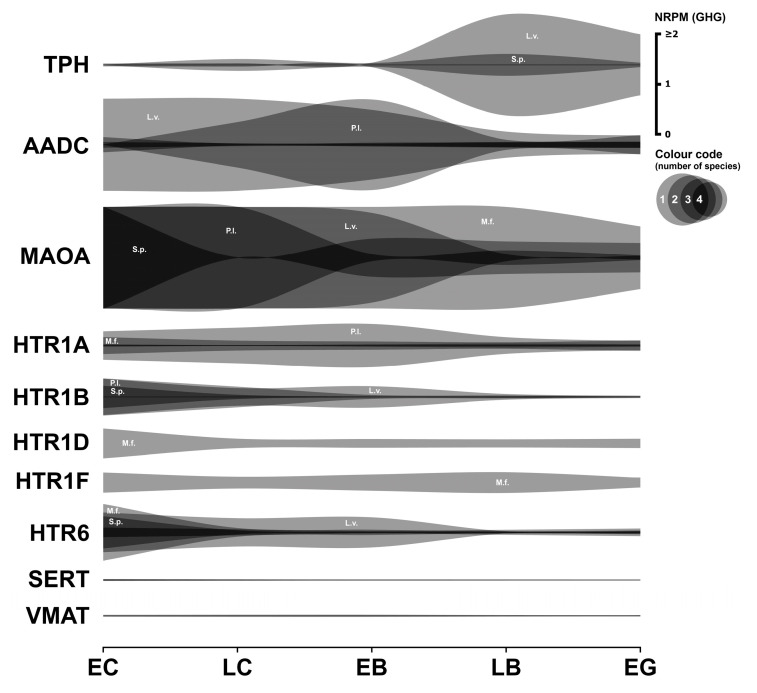
Expression dynamics of genes of serotonergic system in early sea urchin embryos. Expression of all genes is mapped on a single scale, expressed in NRPM—RPM, normalized to geometric mean of three housekeeping genes (GHG). Vertical width of colored zone represents range of NRPM values. Precise numerical data can be found in Supplementary Materials (Table S1). Developmental stages: EC—early cleavage, LC—late cleavage, EB—early blastula, LB—late (mesenchyme) blastula, EG—early gastrula (see Table 1).

Early pharmacological experiments first elucidated the functional significance of the serotonergic system in early sea urchin development [4]. However, the existence of at least 14 distinct serotonin receptor types introduces considerable complexity. This section describes the expression analysis of key components of this system: enzymes controlling serotonin synthesis and degradation, various serotonin receptors, and serotonin transporters (Figure 1). Numerical data are provided in the Supplementary Materials (Table S1).

#### 3.1.1. Serotonergic Enzymes

Serotonin biosynthesis depends on two consecutive enzymatic reactions catalyzed by tryptophan 5-hydroxylase (TPH) and aromatic L-amino acid decarboxylase (AADC). TPH mediates the first and rate-limiting step in this metabolic pathway. In *S. purpuratus*, *TPH* expression increased from undetectable levels during early cleavage to 0.22 GHG (geometric mean of the expression of three housekeeping genes—see Section 2.2 for explanation) during the late cleavage phase, maintaining consistent expression through early gastrulation. Transcripts corresponding to TPH1 isoform X2, matching the *S. purpuratus* genome sequence [23], are expressed during early development in *L. variegatus*. Expression levels increase during late blastula and gastrulation. In contrast, *TPH* transcripts were not detected in the transcriptomes of *M. franciscanus* and *P. lividus* during the early developmental stages.

*AADC*, which catalyzes the second step of serotonin synthesis, shows significant expression in *M. franciscanus*, *S. purpuratus*, and *L. variegatus*. *P. lividus* also exhibits a marked increase in *AADC* expression at the early blastula stage, which contrasts with the low levels observed at the preceding stage and warrants further investigation.

The observed restricted or complete lack of *TPH* transcript expression in three of the four species studied suggests that de novo serotonin synthesis may be significantly limited or potentially absent during the early stages of sea urchin embryogenesis examined here.

Monoamine oxidase (MAO) is the primary enzyme responsible for serotonin and catecholamine degradation. *MAOA* shows high expression levels in all four species (above 1 GHG). However, the dynamic patterns differ considerably: In *M. franciscanus*, high *MAOA* expression is sustained throughout the entire examined period. In *L. variegatus*, it persists until the late blastula stage. In *P. lividus*, expression extends to the early blastula stage, increasing again at late blastula. In *S. purpuratus*, expression is high until late cleavage, increasing again towards the early blastula stage. Despite these differences in dynamic expression patterns, *MAOA* is consistently expressed throughout early development in all four species, suggesting conservation of this monoamine degradation mechanism.

#### 3.1.2. Serotonergic Receptors

Several serotonin receptor transcripts are expressed at significant levels during early sea urchin development. The *HTR6* transcript exhibits the most consistent dynamic expression pattern and maintains high expression levels in all four species, with *M. franciscanus* showing over 1 GHG. *HTR6* expression typically peaks at the egg cell stage, then decreases during the blastula stages, and shows a slight increase at later developmental time points.

*HTR1B* expression is remarkably high in three species (between 0.4 and 0.7 GHG), but negligible in *M. franciscanus*. Conversely, *HTR1D* and *HTR1F* transcripts are significantly expressed only in *M. franciscanus*, showing minimal expression at the blastula stage and increasing during subsequent larval stages.

*HTR1A* is highly expressed in *M. franciscanus* and *P. lividus* from the egg cell stage. In contrast, expression is low or negligible in *S. purpuratus* and *L. variegatus*, indicating significant inter-species differences in receptor expression profiles.

Some other serotonin receptor mRNAs initiate significant expression only at later developmental stages, a pattern likely attributed to MZT.

#### 3.1.3. Serotonergic Transporters

Serotonin transporter (*SERT*)-like sequences were previously identified in developing *P. lividus* using RT-PCR [11]. Furthermore, pharmacological studies have shown that 5-HT reuptake inhibitors, such as imipramine, exert embryophysiological effects in sea urchin embryos [31]. Surprisingly, despite documented transcripts for acetylcholine and noradrenaline transporters (discussed in subsequent sections), no specific 5-HT transporter mRNA was detected in the analyzed *M. franciscanus* transcriptome data. In *P. lividus*, *SERT* expression becomes significant only at the late blastula stage. Conversely, *SERT* is significantly expressed in *L. variegatus* and *S. purpuratus* during cleavage divisions, then drops to near zero around hatching, followed by a six-fold increase during the larval stage.

The vesicular monoamine transporter (*VMAT2*) shows high expression levels in the earliest developmental stages of *L. variegatus*. It is detectably expressed in *S. purpuratus* from the gastrulation stage but remains at a negligible level in *P. lividus*. This suggests that VMAT2-mediated vesicular transport of serotonin is likely not a significant process during early development of *P. lividus*.

#### 3.1.4. Summary

The expression of serotonin receptor mRNAs detected in this analysis confirms previous RT-PCR data and embryophysiological evidence for an effective serotonergic signaling system in early sea urchin embryos. Moreover, similar to mouse embryos [32], some sea urchin species simultaneously express mRNAs of several 5-HT receptor subtypes. This allows the transmitter to participate concurrently in cellular processes such as cytocortex state regulation and interblastomeric signaling.

Conversely, the serotonin synthetic cascade appears to be largely ineffective during early development in sea urchin embryos, based on transcriptome data. In the absence of de novo serotonin synthesis, sea urchin eggs released into seawater likely rely on maternally accumulated transmitter during oogenesis, a situation also observed in mouse embryos and presumably in clawed frogs [33]. Concurrently, the extremely high expression of the serotonin-degrading enzyme *MAOA* suggests that protecting the embryo from excessive transmitter exposure is critically important.

Finally, the lack of significant expression for transcripts encoding serotonin transporters presents a complex and controversial finding. This is surprising given the extensive data on the effects of serotonin reuptake blockers, such as imipramine, on blastomere interactions in early sea urchin embryos [31]. Furthermore, *SERT* mRNA expression was previously detected in *P. lividus* embryos using RT-PCR [11]. This discrepancy suggests either that the transcriptome data or the RT-PCR data may be incomplete, or that imipramine’s effects are mediated not solely through SERT but through another component of the serotonergic regulatory cascade. Another possibility is the involvement of other transporters in this process, beyond SERT and VMAT.

### 3.2. Dopaminergic System

**Figure 2:**
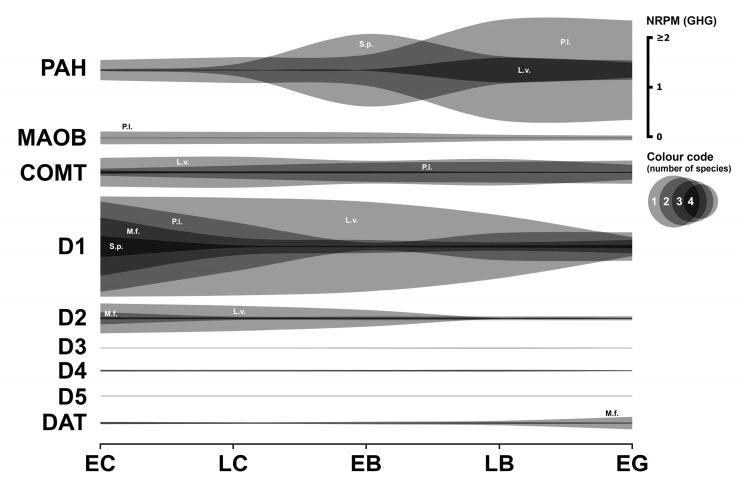
Expression dynamics of dopaminergic system genes in early sea urchins’ embryos. Expression of all genes is mapped to a single scale expressed in NRPM—RPM normalized to geometric mean of three housekeeping genes (GHG). Vertical width of colored zone represents range of NRPM values. Precise numerical data can be found in Supplementary Materials (Table S2). Developmental stages: EC—early cleavage, LC—late cleavage, EB—early blastula, LB—late (mesenchyme) blastula, EG—early gastrula (see Table 1).

This section describes the expression analysis of key components of the dopaminergic system in early sea urchin embryos, including enzymes involved in dopamine synthesis and degradation, as well as dopamine receptors and dopamine transporters (Figure 2). Numerical data are provided in Supplementary Table S2.

#### 3.2.1. Dopaminergic Enzymes

A notable observation is the absence of detectable mRNA transcripts for tyrosine hydroxylase (TH) across all four species’ transcriptomes. Regarding phenylalanine hydroxylase (PAH), *L. variegatus* shows a distinct expression profile: significant expression is detected at fertilization, remains stable during cleavage, and then sharply increases (by over an order of magnitude) at the late blastula stage. This increase may coincide with the onset of the major MZT wave and hatching enzyme synthesis. A similar pattern, characterized by a strong increase at the blastula stage, is observed in *P. lividus*. While *S. purpuratus PAH* expression also increases at the blastula stage, this rise occurs earlier than in *P. lividus*. Although corresponding data are unavailable for *M. franciscanus*, the observed *PAH* mRNA expression levels in the other species suggest the likely presence of a functional PAH enzyme during early sea urchin development.

Catechol-O-methyltransferase (COMT), an enzyme involved in dopamine degradation, is expressed in *M. franciscanus* during cleavage divisions (approximately 0.06 GHG), with a further increase at the pluteus stage. This finding, coupled with the previously discussed high MAO activity (Section 3.1.1), suggests that MAO is likely the predominant pathway for 5-HT and catecholamine degradation during early *M. franciscanus* development. Similar *COMT* expression levels were observed in *S. purpuratus*. In contrast, *COMT* mRNA expression in *L. variegatus* and *P. lividus* is approximately an order of magnitude higher than in *M. franciscanus* and *S. purpuratus*. This difference suggests that COMT-mediated degradation of transmitters may play a more prominent role in *L. variegatus* and *P. lividus* during early embryonic development.

#### 3.2.2. Dopaminergic Receptors

The transcript for the *D1*-like dopamine receptor in *M. franciscanus* is most highly expressed during the earliest developmental stages, reaching minimum expression at the blastula stage. High expression levels of this mRNA and similar dynamic patterns are also observed in the other species, strongly suggesting the presence of a functional D1 receptor protein.

*D4* receptor transcripts are expressed to varying degrees in all species except *M. franciscanus*. *D2* receptor mRNA expression is high in *L. variegatus* and *M. franciscanus* (approximately 0.2–0.6 GHG), comparatively low in *P. lividus*, and close to the significance threshold in *S. purpuratus*.

Transcripts for *D3*-like and *D5*-like dopamine receptors are expressed at very low or negligible levels in *M. franciscanus* and were not detected in the transcriptomes of the other three species.

#### 3.2.3. Dopaminergic Transporter

mRNA sequence fragments corresponding to the sodium-dependent dopamine transporter (DAT) are expressed at significant levels from fertilization in all species studied. This indicates that dopamine reuptake mechanisms are likely present and functional during the earliest developmental stages.

#### 3.2.4. Summary

In summary, the major components of the dopaminergic system (summarized in Figure 2) are transcriptionally represented in early sea urchin embryos, though with significant species-specific differences, particularly in the expression of synthetic enzymes and certain receptor subtypes.

In contrast to serotonin, de novo dopamine synthesis appears possible in early sea urchins, albeit with some limitations. However, a controversial result emerged regarding the dopamine transporter: while its mRNA expression was present in the transcriptome throughout early development, RT-PCR of *P. lividus* showed no *DAT* expression until the pluteus stage [11]. Concurrently, we found that *D1*-like receptor transcript expression is highly conserved, occurring at high levels in all four species. *D2* receptor expression, conversely, is present in two species, consistent with our data on dopamine antagonist effects and RT-PCR data during the cleavage division period [11,34]. In general, the expression pattern of other components resembles that of the serotonergic system in at least three of the four species studied (excluding *M. franciscanus*), indicating a balance between dopamine synthesis and degradation within cells.

Comparative studies in invertebrates (including sea urchins) demonstrate dopamine’s conserved role in larval morphogenesis [35,36]. A specific dopaminergic structure was purportedly found to be involved in the ciliary motility mechanism of swimming sea urchin blastulae. This structure contains dopamine and the D1 dopamine receptor homologue, which were strongly co-localized in 1–2 micrometer diameter granules [10,37]. Our transcriptomic analysis now provides a molecular basis for these phenotypic effects and reveals stage-specific receptor expression windows that may control dopamine sensitivity. Treatment of sea urchin gastrulae with dopamine leads to shortening of the body and post-oral pluteus arms [38]. As in the serotonergic system, early dopaminergic neuronal cells appear in sea urchin embryos as early as the gastrula stage [39].

### 3.3. Adrenergic System

**Figure 3:**
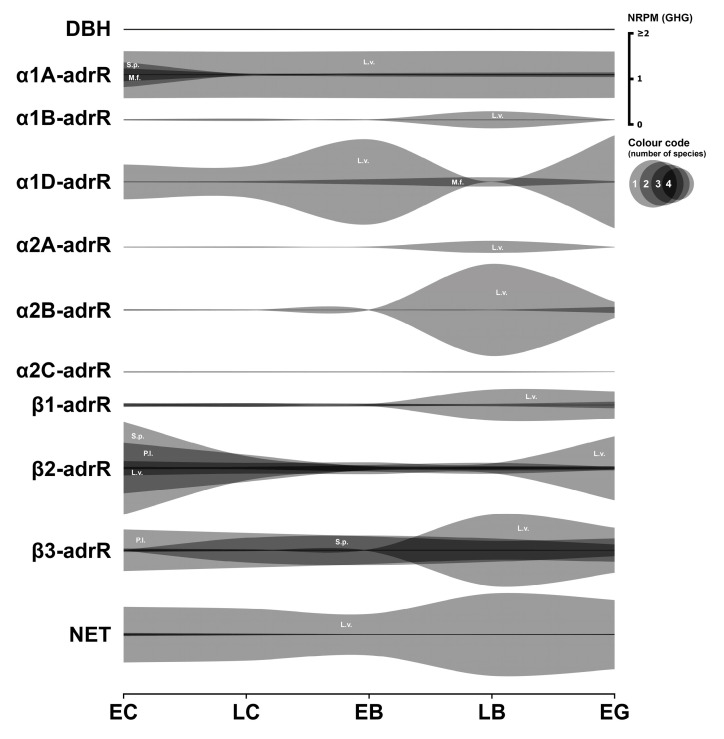
Expression dynamics of adrenergic system genes in early sea urchins’ embryos. Expression of all genes is mapped to a single scale expressed in NRPM—RPM normalized to geometric mean of three housekeeping genes (GHG). Vertical width of colored zone represents range of NRPM values. Precise numerical data can be found in Supplementary Materials (Table S3). Developmental stages: EC—early cleavage, LC—late cleavage, EB—early blastula, LB—late (mesenchyme) blastula, EG—early gastrula (see Table 1).

This section presents the analysis of the expression of key components of the adrenergic system in early sea urchin embryos. The focus is on the enzyme responsible for noradrenaline synthesis, various adrenergic receptor subtypes (α and β), and the noradrenaline transporter (Figure 3). Numerical data are provided in Supplementary Table S3.

#### 3.3.1. Adrenergic Enzymes

Dopamine-β-hydroxylase (DBH), the enzyme that catalyzes the conversion of dopamine to noradrenaline, exhibits barely detectable mRNA expression levels in all species examined, with the exception of *P. lividus*, where levels reach approximately 0.02 GHG. This observation strongly suggests that de novo noradrenaline synthesis likely does not occur at physiologically significant levels during the cleavage stages in most of these species.

#### 3.3.2. Adrenergic Receptors

The expression data for the different subtypes of the α-adrenergic receptor are heterogeneous and appear incomplete across the datasets. Frequently, expression of one or more subtypes is not detectable in certain species, suggesting substantial interspecies differences in the α-adrenergic receptor repertoire.

The *α1A-adrR* transcript is significantly expressed in *M. franciscanus* starting from the egg cell stage (above 0.2 GHG). Expression is also remarkably high in *L. variegatus* and *S. purpuratus*, especially during early developmental stages, but appears to be absent in *P. lividus*.

*α1B-adrR* expression is low in *L. variegatus* and negligible in *M. franciscanus*. In contrast, the α1D-adrenergic receptor-like transcript shows significant expression during early development in both *M. franciscanus* and *L. variegatus*. *α2A-adrR* mRNA was detected exclusively in the transcriptome of *L. variegatus*. Transcripts for the α2B-adrenergic receptor are significantly expressed in *M. franciscanus* and *L. variegatus* from the earliest developmental stages examined, with *L. variegatus* showing a marked increase in expression at the late blastula stage. The α2C-adrenoreceptor-like transcript in *M. franciscanus* is significantly expressed from the 8-blastomere stage and then drops to almost zero by the gastrula stage. The *β1-adrR* transcript shows marked expression during the early developmental stages of *M. franciscanus*, followed by increased expression at the gastrula stage. This receptor sequence is also significantly expressed in *L. variegatus* and *S. purpuratus* after fertilization, but is absent in *P. lividus*. The *β2-adrR* transcript in *M. franciscanus* is moderately but significantly expressed at the earliest developmental stages and shows an order of magnitude increase at the blastula stage. Higher expression levels of the analogous transcript were identified in the other three species, equal to or significantly higher than 1 GHG. The *β3-adrR* transcript in *M. franciscanus* is moderately expressed after fertilization and subsequently decreases. In *L. variegatus*, the expression level is an order of magnitude higher and shows a strong increase at the hatching stage. In *P. lividus*, the expression level approaches 1 GHG. The situation is less clear for *S. purpuratus*; here, expression at fertilization is close to the significance limit, followed by a substantial increase during late cleavage.

#### 3.3.3. Adrenergic Transporter

The *M. franciscanus* sodium-dependent norepinephrine transporter (NET)-like isoform X1 mRNA is only significantly expressed from the larval stage onwards in the examined dataset. *NET* is expressed in moderate but significant amounts in *P. lividus* and *S. purpuratus*, while it reaches very high expression levels (above 1 GHG) in *L. variegatus*.

#### 3.3.4. Summary

The adrenergic system components represented at the mRNA level in early sea urchin development encompass a variety of receptors with diverse expression patterns across species. The presence of functional β-receptors appears most probable, given their more consistent and often robust expression across the species examined. It is noteworthy that data on the presence of NET in *P. lividus* embryos are consistent with our previous RT-PCR results, which showed the presence of a single transmitter transporter in early embryos [11]. However, the overall functional significance of this system during early cleavage remains somewhat enigmatic, particularly considering the limited transcriptomic evidence for endogenous norepinephrine synthesis.

### 3.4. Cholinergic System

**Figure 4:**
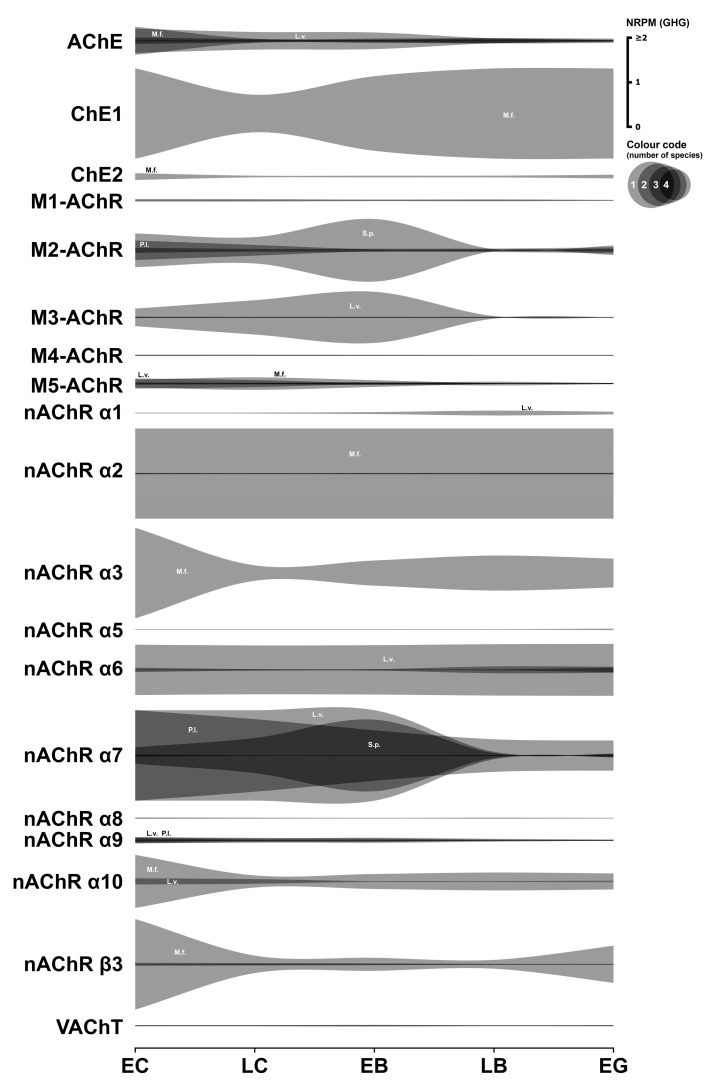
Expression dynamics of cholinergic system genes in early sea urchins’ embryos. Expression of all genes is mapped to a single scale expressed in NRPM—RPM normalized to geometric mean of three housekeeping genes (GHG). Vertical width of colored zone represents range of NRPM values. Precise numerical data can be found in Supplementary Materials (Table S4). Developmental stages: EC—early cleavage, LC—late cleavage, EB—early blastula, LB—late (mesenchyme) blastula, EG—early gastrula (see Table 1).

This section details the expression analysis of key components of the cholinergic system in early sea urchin embryos. These include enzymes for acetylcholine (ACh) synthesis and degradation, muscarinic and nicotinic ACh receptors, and the vesicular acetylcholine transporter (VAChT). Transcriptome data for this system are summarized in Figure 4. Numerical data are presented in Supplementary Table S4.

#### 3.4.1. Cholinergic Enzymes

Choline-O-acetyltransferase (ChAT), crucial for ACh synthesis, was undetected in *M. franciscanus* transcriptome data and exhibited negligible expression in *L. variegatus*. Despite ACh’s established developmental roles, the absence of *ChAT* transcripts is puzzling, suggesting that ACh may be synthesized and maternally stored in gametes prior to fertilization.

Three distinct annotated cholinesterase mRNAs were identified in *M. franciscanus*. Notably, acetylcholinesterase (AChE) mRNA transcripts were significantly expressed from the egg cell stage, subsequently decreasing in all four species examined. This pattern aligns with immunohistochemical localization and previous findings [40].

#### 3.4.2. Cholinergic Receptors

Among muscarinic acetylcholine receptor transcripts, the *M2*-like transcript showed the highest expression levels (approximately 0.1 to 0.7 GHG) across all species, except *L. variegatus*, where it was notably absent. *M3-AChR*-like transcripts (absent in *P. lividus*) and *M5*-like transcripts (absent in *S. purpuratus*) also exhibited significant expression in three out of the four species.

*M1-AChR*-like transcript expression was detected solely in *L. variegatus* during early development, while *M4-AChR* expression was observed only in *M. franciscanus*.

Several subunits of the nicotinic AChR (nAChR) complex are expressed during the early development of the studied sea urchin species. Functional nAChRs typically involve the assembly of α and β subunits, or can form homomeric receptors solely composed of α7 subunits [41,42,43].

Heteromeric receptors (containing both α and β subunits) appear to be a possible structural variant in *M. franciscanus* and *P. lividus*, where transcripts for various α-subunits are co-expressed with the β3-subunit transcript. In *M. franciscanus*, the β3-subunit transcript is highly expressed from the egg cell stage (2.9 GHG), declines to a minimum at the blastula stage, and subsequently increases during larval stages. The expressed α-subunit repertoire in *M. franciscanus* includes *nAChR α2* (38 GHG), *nAChR α3* (5203 GHG), *nAChR α7* (0.035 GHG), and *nAChR α10* (1167 GHG). Although β3-subunit expression in *P. lividus* is considerably lower than in *M. franciscanus*, it remains significant (0.05 GHG) and co-occurs with expression of several α-subunits (*nAChR α6*: 0.004 GHG, *nAChR α7*: 2 GHG, and *nAChR α9*: 0.1 GHG).

The apparent absence of detectable β-subunit expression during early development in *L. variegatus* and *S. purpuratus* questions the formation of canonical heteromeric nAChRs in these species during this period. Nonetheless, transcripts for several α-subunits are expressed in their respective transcriptomes. Significant expression of nAChR subunits α1, α2, α6, α7, α9, and α10 mRNAs was detected in *L. variegatus*. In *S. purpuratus*, the expressed α-subunits include α6, α7, α8, and α9.

The α7 receptor represents a homomeric nAChR subtype composed of five identical α7 subunits [41,42,43]. Such receptors constitute another plausible configuration for functional nAChRs in sea urchin embryos. Significantly, transcripts for this α7 subunit were detected in substantial amounts during development across all four species. In *P. lividus* and *L. variegatus*, α7 expression reached exceptionally high levels, considerably exceeding the GHG normalization factor. Crucially, the *nAChR α7* is the only nAChR subunit whose mRNA expression was consistently detected at significant levels across all four species examined. Furthermore, previous immunological studies identified the nAChR α7 protein during fertilization in *P. lividus* [40].

Collectively, the transcriptome data support the potential formation of various structural types of nicotinic ACh receptors in early sea urchin embryos.

#### 3.4.3. Cholinergic Transporter

In *M. franciscanus*, the only identified mRNA transcript for the vesicular acetylcholine transporter (VAChT) exhibited negligible or absent expression until the late gastrula stage. Conversely, *VAChT* expression in *L. variegatus* was significant across all early stages examined.

#### 3.4.4. Summary

In summary, diverse cholinergic system components are transcriptionally expressed during early sea urchin development, with species-specific variations. Notable exceptions, present at relatively high transcriptional levels across all species, include the α7 subunit of nAChR and the enzyme AChE. Acetylcholine is known to influence early sea urchin embryo development from fertilization, with effects varying by developmental stage and receptor type, as demonstrated in *P. lividus* [44]. Cholinergic mechanisms participate in regulating intracellular Ca^2+^ levels and protein kinase C activity [17,45]. These findings underscore the sensitivity of early embryos to acetylcholine. The functional implications of the observed interspecies differences in cholinergic component expression warrant further investigation.

### 3.5. GABAergic System

**Figure 5:**
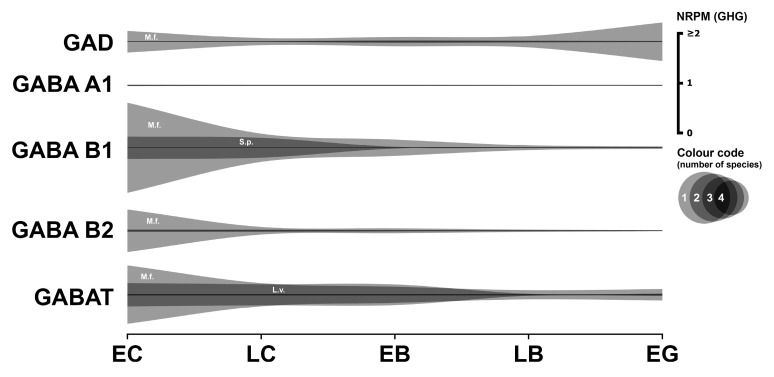
Expression dynamics of GABAergic system genes in early sea urchins’ embryos. Expression of all genes is mapped to a single scale expressed in NRPM—RPM normalized to geometric mean of three housekeeping genes (GHG). Vertical width of colored zone represents range of NRPM values. Precise numerical data can be found in Supplementary Materials (Table S5). Developmental stages: EC—early cleavage, LC—late cleavage, EB—early blastula, LB—late (mesenchyme) blastula, EG—early gastrula (see Table 1).

Information regarding the influence of the GABAergic system on early sea urchin development is extremely limited. The available transcriptome data concerning this system are summarized in Figure 5. Numerical data are provided in Supplementary Table S5.

#### 3.5.1. GABAergic Enzyme

Glutamate decarboxylase (GAD), specifically the identified fragment of isoform X2 in *M. franciscanus*, is significantly expressed from fertilization (0.4 GHG). Expression decreases during the blastula stages before increasing again to 0.75 GHG at the gastrula stage. Significant *GAD* mRNA expression is also observed in *S. purpuratus*, but only from the early blastula stage.

#### 3.5.2. GABAergic Receptors

Expression of ionotropic *GABA A* receptor subunits is present at low levels in early *S. purpuratus* embryos and only in trace amounts in developing *L. variegatus*. No data for this receptor were found in the transcriptomes of the other two species.

The metabotropic GABA B receptor is known to function as an obligate heterodimer, requiring the co-assembly of B1 and B2 subunits for proper signaling [46]. The effects of GABAergic compounds on early sea urchin development are currently undocumented in the literature. However, our transcriptome analysis shows relatively high expression levels of *GABAB* receptor subunits during early development in *M. franciscanus* and *S. purpuratus*.

The *GABA B1*-like fragment of *M. franciscanus* exhibits high expression at the egg cell stage (1.778 GHG), which subsequently decreases more than threefold, reaching a minimum at the blastula stage. The expression of this receptor mRNA in *S. purpuratus* is also significant from the egg cell stage (0.4373 GHG) and then declines. The *GABA B2*-like mRNA fragment of *M. franciscanus* is also highly expressed at the earliest stage (0.829 GHG), and considerable expression is observed in *S. purpuratus*; however, no data are available for the other two species. The expression pattern for *GABA B2* in both *M. franciscanus* and *S. purpuratus* is characterized by maximal expression at the beginning of development (egg cells and cleavage divisions), followed by a gradual decline to levels close to zero. Therefore, the formation of functional GABA B receptors appears likely in both *M. franciscanus* and *S. purpuratus*.

#### 3.5.3. GABAergic Transporter

The GABA transporter 2-like fragment is highly expressed in *M. franciscanus* and *L. variegatus* from the egg cell stage (1.15 and 0.45 GHG, respectively), with expression levels decreasing thereafter.

#### 3.5.4. Summary

In summary, the available transcriptomic data concerning the GABAergic system are currently limited, precluding definitive conclusions about its comprehensive presence and specific roles during early sea urchin development (Table S5). The increase in *GAD* expression at the gastrula stage coincides with the emergence of GABAergic neural progenitors [47], suggesting dual roles for GABA—first in pre-nervous development, later in neurogenesis. While our study focuses on pre-nervous stages, we note that serotonin, dopamine, and GABA jointly regulate locomotory behavior in pluteus larvae [48], suggesting functional continuity of these systems throughout development.

### 3.6. Histaminergic System

**Figure 6:**
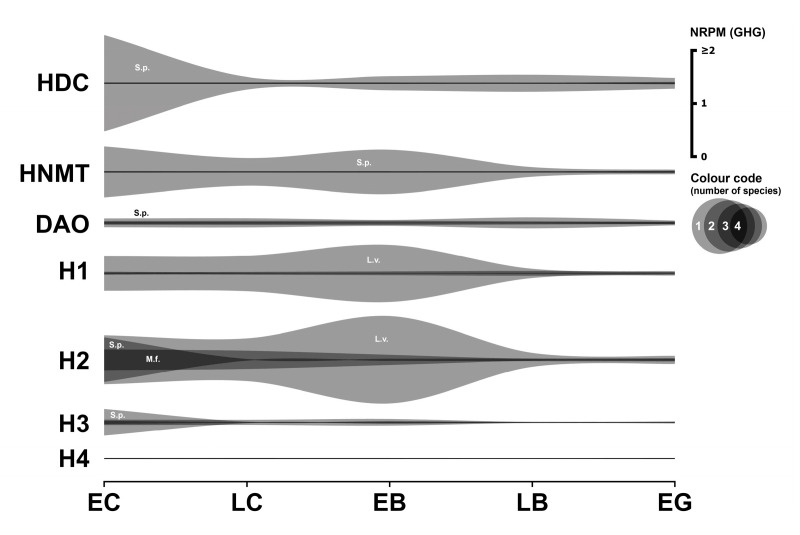
Expression dynamics of histaminergic system genes in early sea urchins’ embryos. Expression of all genes is mapped to a single scale expressed in NRPM—RPM normalized to geometric mean of three housekeeping genes (GHG). Vertical width of colored zone represents range of NRPM values. Precise numerical data can be found in Supplementary Materials (Table S6). Developmental stages: EC—early cleavage, LC—late cleavage, EB—early blastula, LB—late (mesenchyme) blastula, EG—early gastrula (see Table 1).

The only documented role of a histaminergic mechanism in early sea urchin development involves the activation of the nitric oxide (NO) signaling pathway via the histamine H1 receptor, leading to NO production and subsequent Ca^2+^ release from intracellular stores [49]. Transcriptome data related to the histaminergic system are summarized in Figure 6. Numerical data are provided in Supplementary Table S6.

#### 3.6.1. Histaminergic Enzymes

Histidine decarboxylase (HDC), the enzyme responsible for histamine synthesis, is highly expressed in *S. purpuratus* from the cleavage divisions (1.78 GHG), after which expression decreases by an order of magnitude. *HDC* expression is insignificant or absent in the transcriptomes of the other three species analyzed.

Two primary enzymes are involved in histamine metabolism: histamine N-methyltransferase (HNMT) and diamine oxidase (DAO). High expression of *HNMT* is found only in *S. purpuratus*, with moderate but significant amounts in *L. variegatus*. *DAO* is significantly expressed in three species but is absent in *M. franciscanus*. The absence of *HDC* and *DAO* in *M. franciscanus* is notable, given the detectable expression of histamine receptors in embryos of this species (see below).

#### 3.6.2. Histaminergic Receptors

Previous RT-PCR analysis detected *H1* receptor mRNA expression in *S. purpuratus* [49]. Our transcriptome analysis reveals significant initial *H1* expression in *S. purpuratus* (0.0447 GHG) that subsequently decreases by an order of magnitude. The *M. franciscanus* histamine *H1* receptor mRNA fragment exhibits relatively constant expression levels during early developmental stages (approximately 0.013 GHG), comparable to *S. purpuratus*. The most pronounced *H1* expression is observed in *L. variegatus* (0.63 GHG). Surprisingly, no traces of any histamine receptor mRNA were found in the transcriptome of *P. lividus*.

The histamine *H2* receptor-like fragment shows significant expression from the egg cell stage in *M. franciscanus* (0.377 GHG), decreasing thereafter by more than 10-fold but remaining above the significance threshold. Expression of histamine *H2* receptor mRNA in *L. variegatus* and *S. purpuratus* is strong at the egg cell stage (approximately 0.8 GHG) and subsequently decreases at the blastula and gastrula stages.

The histamine *H3* receptor-like mRNA fragment exhibits its strongest expression in *S. purpuratus* (0.483 GHG) and significantly lower expression levels in *M. franciscanus* and *L. variegatus* at the first developmental stage examined. The *H4* receptor-like mRNA fragment was found only in small or trace amounts in all species.

#### 3.6.3. Histaminergic Transporter

No traces of expression of histamine transporters were found in any of the species examined, including VMAT2, which can transport histamine in some systems (see Section 3.1.1, Serotonergic system, Transporters, for discussion of VMAT2).

#### 3.6.4. Summary

In summary, mRNAs encoding three types of histamine receptors (*H1*, *H2*, and *H3*) are expressed in significant amounts across all species examined, with the notable exception of *P. lividus* (Table S6). The established role of histamine in both sea urchin fertilization and subsequent larval metamorphosis [50] suggests that histamine sensitivity may be a conserved requirement throughout embryonic development.

### 3.7. Glutamatergic System

**Figure 7:**
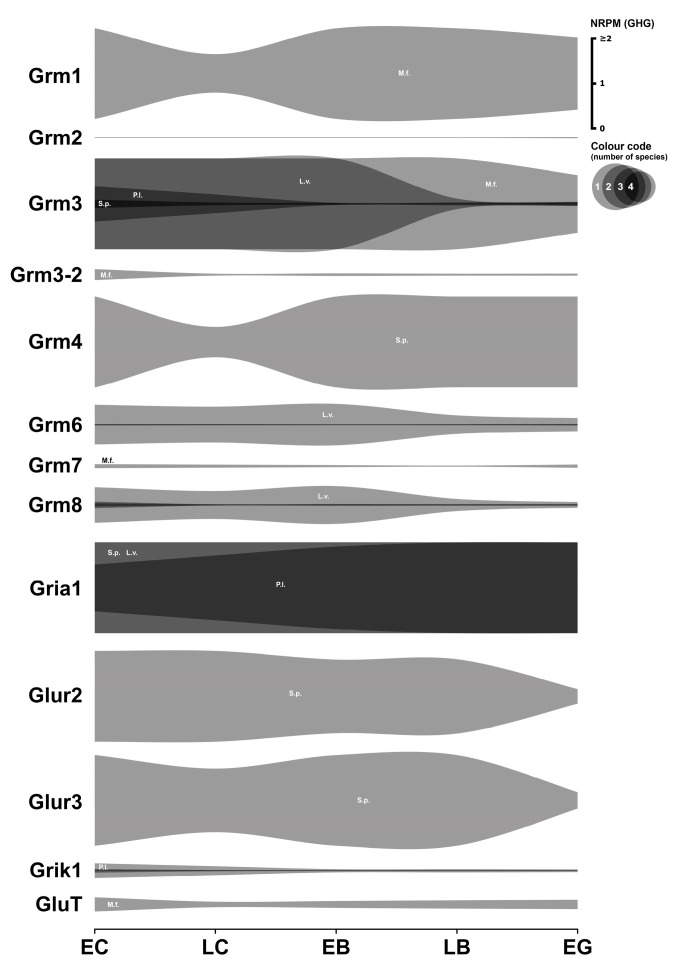
Expression dynamics of glutamatergic system genes in early sea urchins’ embryos. Expression of all genes is mapped to a single scale expressed in NRPM—RPM normalized to geometric mean of three housekeeping genes (GHG). Vertical width of colored zone represents range of NRPM values. Precise numerical data can be found in Supplementary Materials (Table S7). Developmental stages: EC—early cleavage, LC—late cleavage, EB—early blastula, LB—late (mesenchyme) blastula, EG—early gastrula (see Table 1).

This section examines the expression profiles of key components of the glutamatergic signaling system in early sea urchin embryos, focusing on metabotropic and ionotropic glutamate receptors and glutamate transporters. All related data are summarized in Figure 7. Numerical data are provided in Supplementary Table S7.

#### 3.7.1. Glutamatergic Receptors

The expression levels observed for metabotropic glutamate receptors are remarkably pronounced in these transcriptomes. The *Grm1* transcript of *M. franciscanus* shows very high initial expression (about 8 GHG), which then decreases dramatically (approximately 5-fold) until the gastrula stage. Neither *Grm1* transcripts in the other three species nor *Grm2* mRNAs in any of the examined species were detected in significant quantity or at all in the transcriptomes.

The *Grm3*-like transcript in *M. franciscanus* has the highest initial expression levels detected in this whole transcriptome analysis—approximately 30 GHG—which subsequently decreases until the gastrula stage. Comparably high expression of *Grm3* mRNA is found in developing *L. variegatus*, where a marked increase occurs at the early blastula stage, followed by a sharp decrease at the late blastula stage. The expression of this mRNA in the other two species (*S. purpuratus* and *P. lividus*) is much lower, although still statistically significant.

*Grm8* mRNA was detected in the developing embryos of three species (*M. franciscanus*, *L. variegatus*, *S. purpuratus*) at levels ranging from 0.05 to 0.77 GHG, but was absent in *P. lividus*. The expression of *Grm6* was quite high in *L. variegatus* and relatively low in *P. lividus*, while it could not be detected in the other two species. Expression of *Grm4* was only detected in *S. purpuratus*, albeit at a significant level. Expression of *Grm7* mRNA was detected exclusively in *M. franciscanus*.

Transcriptome data on the expression of ionotropic glutamate receptors are comparatively sparse. In *M. franciscanus*, transcripts for kainate receptors are only expressed from the prism stage onwards, while AMPA and NMDA receptor transcripts were not detected at all in the analyzed early developmental stages. At the same time, in *P. lividus*, *L. variegatus*, and *S. purpuratus*, mRNAs encoding subunits of AMPA and kainate receptors are expressed at extremely high levels—between 1 and 16 GHG during early development.

#### 3.7.2. Glutamatergic Transporter

Expression of the cystine/glutamate transporter isoform X1 was only detected in *M. franciscanus*, where it reached quite high levels; it was not present in the transcriptomes of the other three species.

#### 3.7.3. Summary

In summary, the glutamatergic system shows very different expression patterns in the different sea urchin species studied. *M. franciscanus* exhibits a unique profile characterized by a high expression of specific metabotropic receptors (*Grm1*, *Grm3*) and the cystine/glutamate transporter, combined with a lack of detectable expression of ionotropic receptors during early development. In striking contrast, *P. lividus*, *L. variegatus*, and *S. purpuratus* show marked expression of AMPA- and kainate-type ionotropic receptors. These significant differences between species suggest that glutamate signaling during early development may play a different physiological role in these related echinoderms. The available transcriptome data pertaining to this system are summarized in Supplementary Table S7.

### 3.8. Spatial Analysis Using Single-Cell Transcriptomic Data

To investigate the spatial organization and potential for co-expression of transmitter system components, we leveraged publicly available single-cell RNA-seq data for *S. purpuratus* [51]. This approach provides valuable spatial information at cellular resolution, which is particularly useful given the technical challenges of performing comprehensive in situ hybridization (ISH) for multiple genes across four different species (addressing R2).

We focused our analysis on the early blastula stage, which precedes the major wave of the MZT. This stage is ideal for assessing the initial distribution of maternally supplied transcripts before large-scale zygotic expression begins. A primary challenge for a comprehensive co-expression analysis is that most transmitter-related genes are expressed at very low levels during these stages. This low abundance limits the statistical power needed to confidently determine if two low-expression genes are truly co-expressed within the same cells.

**Figure 8:**
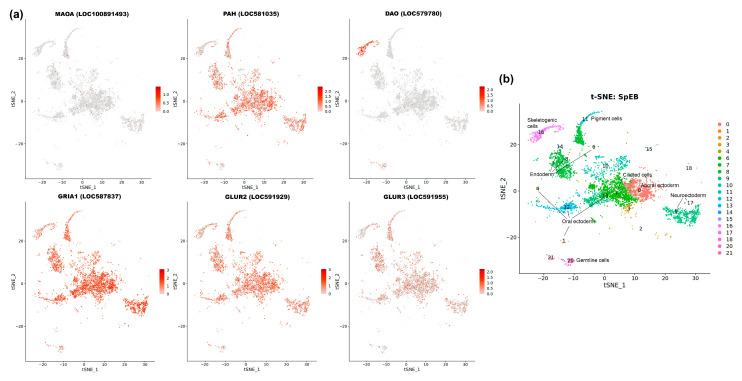
Single-cell transcriptomic analysis of transmitter-related gene expression in the *S. purpuratus* early blastula stage. (**a**) Feature plots showing expression of *MAOA*, *PAH*, *DAO*, *GRIA1*, *GLUR2*, and *GLUR3*, which are among the most highly expressed transmitter-related genes at this stage. Cells are colored by relative expression level. (**b**) t-SNE plot of early blastula stage, visualizing the 20 distinct cell clusters that were detected. Corresponding cell populations are colored by cluster identity and annotated on the plot according to [51].

Despite this limitation, the analysis was highly informative for the most abundant transmitter-related genes at this stage: *MAOA*, *PAH*, *DAO*, *GRIA1*, *GLUR2*, and *GLUR3* (Figure 8). The results revealed two distinct patterns. Firstly, we observed a notable enrichment of *DAO* transcripts in cluster 6, corresponding to the future skeletogenic primary mesenchyme cells (PMCs). This is a key finding, demonstrating an early lineage-specific localization of a component involved in amine metabolism. Secondly, the other analyzed genes (*MAOA*, *PAH*, and the glutamate receptors) showed a broad and uniform distribution across all embryonic clusters, including the germline cells.

This uniform pattern is not a lack of signal, but rather a significant biological result. It strongly supports the hypothesis that these transcripts are part of a maternally loaded reservoir, evenly distributed throughout the oocyte and persisting in all cells of the early embryo. Thus, while the data indicate that robust, cell-type specific co-expression networks for these systems have not yet formed, they provide critical spatial evidence that the embryo is equipped with a ubiquitous, pre-patterned supply of transmitter-related mRNAs well before the appearance of the first neurons.

## 4. Discussion

Our study employed a comparative transcriptomic approach using four species of regular sea urchins: *Mesocentrotus franciscanus*, *Lytechinus variegatus*, *Paracentrotus lividus*, and *Strongylocentrotus purpuratus*. These species were chosen for two main reasons: the availability of high-quality, stage-specific transcriptomic datasets, and their representation as members of the Euechinoidea sub-class, which includes many widely used model organisms in developmental biology. Phylogenetically, these species offer broad taxonomic coverage, spanning three distinct families: Strongylocentrotidae (*S. purpuratus* and *M. franciscanus*), Parechinidae (*P. lividus*), and Toxopneustidae (*L. variegatus*). This diversity allowed us to investigate potential correlations between expression patterns and evolutionary relationships. However, our analysis did not reveal clear phylogenetic trends in the data. Given this finding, our study focused on identifying conserved properties and fundamental principles of transmitter system expression common to all four species.

Our comparative analysis demonstrates that mRNAs encoding core components of seven neurotransmitter systems—serotonergic, dopaminergic, adrenergic, cholinergic, glutamatergic, GABAergic, and histaminergic—are present and dynamically regulated from fertilization through gastrulation. To enable direct comparison across species and provide a clear developmental context, we harmonized the developmental stages using a unified nomenclature (Table 1). The analysis begins with early cleavage, representing initial cell divisions shortly after fertilization, and progresses to late cleavage, embryos composed of tens of cells. This is followed by the early blastula, a non-epithelialized embryo, and the late blastula, characterized by the ingression of primary mesenchyme cells and motility. This latter stage is particularly critical as it encompasses both hatching and the major wave of zygotic genome activation. The final stage examined, the early gastrula, is marked by the onset of major morphogenetic movements, including the initiation of invagination. Our findings indicate that dynamic regulation of these transmitter systems occurs across all these stages, well before the emergence of neural structures.

A significant challenge in early developmental transcriptomics is data normalization. Normalization of transcriptomic data was performed relative to the geometric mean of the expression levels of three housekeeping genes: Glyceraldehyde-3-phosphate dehydrogenase (*GAPDH*), ornithine decarboxylase (*ODC*), and hypoxanthine-guanine phosphoribosyltransferase (*HPRT*). These genes are well-established reference genes frequently used in developmental studies [52,53]. As a practical prerequisite for comparative analysis, they were robustly expressed and clearly annotated in all four transcriptome datasets examined. Additionally, to experimentally validate their suitability, we leveraged publicly available single-cell RNA-seq data from *S. purpuratus* [51]. This analysis confirmed that all three selected housekeeping genes exhibit broad, ubiquitous expression across all major cell clusters (Figure S1). This is a critical characteristic for a reliable housekeeping gene, as it demonstrates that their expression is not biased towards any specific developing cell lineage, thus making them suitable for global normalization of the whole-embryo transcriptome.

To provide evidence for the effectiveness of our normalization strategy, we assessed whether the resulting data accurately reflect known biological phenomena. Following normalization, our data show a clear, widespread increase in the transcript abundance of numerous genes at the blastula stage across all four species. This dynamic pattern precisely corresponds with the well-documented timing of the major wave of MZT, a fundamental and highly conserved event in early sea urchin development. The ability of our housekeeping gene-based normalization to clearly resolve this key developmental transition serves as strong internal validation. The calculated geometric mean values used for normalization at each stage are provided in Table 2. Normalization of transcriptome data using appropriate housekeeping genes refines the interpretation of mRNA expression dynamics and enables more meaningful comparisons between different species. For example, normalized *HTR6* expression clearly peaks in the unicellular embryo and then declines sharply (Figure 1), a pattern possibly indicative of rapid mRNA consumption driving protein synthesis early on. Conversely, the expression of *HTR1A* remains consistently low in the early stages studied in certain species (Figure 1), while the expression of enzymes such as phenylalanine hydroxylase increases markedly during the period corresponding to the major wave of MZT (Figure 2).

In general, the most common pattern of such dynamics after data normalization is a relatively high initial level of mRNA at the one-cell stage, followed by a gradual decrease in expression during the blastula stage, and then an increase up to the gastrula stage, logically associated with the activation of the zygotic genome. In parallel, our studies yielded a result that challenges the widespread assumption of general transcriptional quiescence prior to the major wave of zygotic genome activation, commonly referred to as the MZT. Initial visualization attempts of mRNA expression dynamics indicated an apparent increase in transcript levels of numerous genes after fertilization, including many encoding components of the neurotransmitter systems described above (Figure 1, Figure 2, Figure 3, Figure 4, Figure 5, Figure 6 and Figure 7). Although there is evidence for early low zygotic transcription in other model systems such as *Ascaris suum* [54] and *Xenopus* [55,56,57,58,59], our initial results prompted a re-evaluation of quantification and normalization approaches in the context of early sea urchin development.

**Figure 9:**
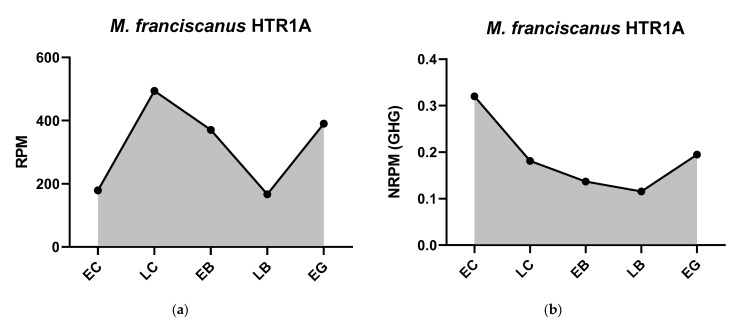
Dynamics of *M. franciscanus HTR1A* mRNA expression. (**a**) Original data in RPM from transcriptome of *M. franciscanus*. (**b**) NRPM data normalized to geometric mean expression of three housekeeping genes (GHG), where GHG is set to 1. Lower *y*-axis scale in (**b**) reflects that receptor’s expression is a small fraction relative to the highly abundant GHGs.

Furthermore, we noted an unexpected apparent increase in the expression levels of canonical housekeeping genes shortly after fertilization. The data in Table 1 reflect the non-normalized values that led to this observation. This apparent increase was widespread, affecting more than half of the sequences examined in some datasets, including transcripts that showed negligible expression at the unfertilized egg stage. The observation that even housekeeping genes, which are generally expected to have relatively stable expression levels, exhibited this pattern raised concerns about the reliability of absolute expression quantification, particularly when comparing between early developmental time points using certain RNA processing or library preparation methods. Interestingly, similar studies in *Xenopus* show that early increases in transcript levels often occur in total RNA samples but not in rRNA-depleted ones (e.g., RiboZero-treated) [58]. This suggests that technical factors—such as RNA extraction, library preparation, or data processing—could influence these patterns. By normalizing our data using the geometric mean of three housekeeping genes (GHG normalization), we obtained expression profiles that better fit the expected gradual decline of maternal mRNA during early development (Figure 9). This decrease likely reflects natural mRNA turnover and ongoing protein synthesis from maternal transcripts. Importantly, our approach still detects probably true expression increases, such as those seen at the late blastula stage, which align with hatching enzyme production and MZT [29].

To firmly establish the connection between the observed gene expression upswing and zygotic genome activation, it is crucial to consider the specific timing of this event in all four species. While the inference is often based on *S. purpuratus*, where the major wave of MZT occurs between the 6th and 7th cleavage (64- to 128-cell stage), marking the transition to the early blastula [28], this timing is highly conserved. Supporting this, large-scale zygotic transcription in both *P. lividus* and *L. variegatus* also commences at the early blastula stage [29]. Although specific high-resolution timing studies are less common for *M. franciscanus*, its developmental program is homologous, and the transition to a blastula of several hundred cells represents the equivalent developmental landmark for the onset of major MZT [24]. Therefore, the widespread increase in transcript abundance at the early blastula (EB) and late blastula (LB) stages that our normalized data reveals across all four species aligns consistently and is strongly supported by the known timing of the major MZT in each lineage.

Our findings promote the relatively new notion of transmitters as universal regulators of a series of parallel and sequential processes throughout individual development from oogenesis to specialized functions and processes of the mature organism, including oncogenesis and nervous transmission [20]. The dynamics of expression of a particular component of the mediator mechanism allows us to understand in more detail how spatially localized mediator systems coordinate cell behavior (e.g., local changes in the properties of the cytocortex, interactions between blastomeres, epithelial folding) without classical synaptic connections. A comparative analysis of the transcriptomes of four sea urchin species sheds light on the conserved and varying elements of the transmitter mechanisms of early development. It indirectly points to the most likely transmitter mechanisms operating during early embryogenesis and, accordingly, to the expression of the receptor and transporter proteins involved in the previously discovered embryonic physiological processes. Transcriptomic data on the expression dynamics of specific mRNAs of transmitter systems, starting from the earliest stages, allow us to correlate them to the above-mentioned processes. The observed variability in some gene expression profiles between species, when viewed through this lens, may reflect the inherent plasticity of transmitter system regulation. These differences could be related to species-specific adaptations or simply reflect the evolutionary distances separating these sea urchin lineages [22].

Co-expression of transcripts for at least two different 5-HT receptor types, including *HTR6* throughout, is detected in each species at the fertilized egg stage. This finding is in good agreement with previous pharmacological evidence demonstrating embryostatic effects of HTR1 and HTR6 antagonists in *P. lividus* embryos [11]. The species-specific nature of the expressed 5-HT receptor subtypes suggests potential functional diversification, and the possibility that multiple functional 5-HT receptor subtypes coexist and act in the same embryonic cell cannot be ruled out.

The high expression of monoamine oxidase mRNA contradicts the previously held view that transmitter release may be the primary and main mechanism for signal inactivation in early embryos. Instead, our data suggest a potentially crucial role of enzymatic degradation in the control of transmitter concentration. This implies the existence of protective mechanisms that prevent excessive or prolonged transmitter signaling that might otherwise interfere with the precise orchestration of normal developmental events.

In the three species studied in which dopamine synthesis appears to be possible (*L. variegatus*, *P. lividus*, *S. purpuratus*), at least two different dopamine receptor subtypes show significant transcript expression. The generally higher expression levels observed for *D1*-like receptors compared to *D2*-like receptors are in some contrast to the known embryostatic effects of haloperidol (a preferential D2 antagonist) in *P. lividus*, strongly supporting the involvement of *D2*-like receptors in mediating these effects [11,34]. The expression of adrenoreceptors, particularly β-receptor subtypes, is also significant in several species. This correlates interestingly with the documented ability of various adrenergic ligands to influence cleavage divisions and modulate cytocortical stiffness [12].

Although the overall expression of muscarinic acetylcholine receptor transcripts appears relatively low compared to some other receptors, the available pharmacological and immunological data provide strong evidence for their functional activity, especially during fertilization. The observed set of expressed nAChR subunits raises interesting questions, especially considering previous evidence of involvement in the processes of sea urchin embryophysiology [40,60,61,62]. High expression of the α7 subunit of nAChR is observed in all species except *M. franciscanus*. The lack of expression of the β-subunit in *L. variegatus* and *S. purpuratus* in the early stages suggests the possibility of the formation of exclusively homomeric α7-nAChRs in these species. Conversely, the exceptionally high expression of the β3 subunit with simultaneous expression of several α-subunits in *M. franciscanus* also suggests the formation of heteromeric nAChRs in this species. Such a strong difference between relatively closely related species, three of which once belonged to the genus *Centrotus*, is surprising, but characterizes the possible extent of species-specific variability. The high expression of acetylcholine-cleaving enzymes (AChE), similar to enzymes that cleave serotonin and catecholamines (MAO, COMT), highlights the likely importance of mechanisms for premature termination of signaling, as excessive or uncontrolled activation of the cholinergic system may impair development.

In addition, significant expression levels were found for GABAB receptors in *M. franciscanus* and *S. purpuratus*, histamine receptors (*H1*, *H2*, and *H3*) in three species (except *P. lividus*), the metabotropic glutamate receptor grm3 (*Grm3*) especially in *M. franciscanus* and *L. variegatus*, and ionotropic AMPA-type glutamate receptors in three species (except *M. franciscanus*). The latter finding of high AMPA receptor expression is particularly unexpected given the lack of embryophysiological data to date suggesting glutamatergic mechanisms in early sea urchin development. However, these transcriptomic findings provide a tantalizing prospect and a clear rationale for initiating pharmacological experiments to investigate the potential role of glutamate signaling in these embryos.

The validation of RNA-seq data against other quantification methods, such as RT-PCR, is important to ensure the extracted biological patterns are meaningful. This is particularly relevant given the potential for discrepancies between datasets. A comparison with published literature provides a clear context for interpreting our results. A prime example is the expression of *SERT* in *P. lividus*. While sensitive methods like RT-PCR have experimentally confirmed its presence in early development [11], it is not detected at a significant level in the bulk transcriptomic data. This discrepancy highlights a fundamental difference in the sensitivity of these methods. RT-PCR is an amplification-based technique capable of detecting very low-copy-number transcripts. In contrast, bulk RNA-seq provides an average expression level across all cells of the embryo. A transcript that is expressed at a low level, or perhaps restricted to only a few cells, can have its signal diluted below the reliable detection threshold in a whole-embryo sample. The discrepancy we previously noted for *DAT* expression in *P. lividus* likely falls into this same category of methodological differences. Despite these limitations for detecting low-abundance transcripts, our comparative transcriptomic approach provides an invaluable advantage that RT-PCR cannot: its ability to offer a broad, systems-level, and comparative perspective simultaneously across seven transmitter systems and four species. Therefore, the primary and robust biological information that can be extracted from our analysis is the conserved presence and dynamic regulation of the majority of components for these systems throughout early, pre-neural development. This strongly suggests a deep molecular overlap between pre-nervous transmitter mechanisms and their definitive counterparts at later life stages, adding weight to the growing understanding that neurotransmitter signaling influences diverse biological processes throughout ontogenesis. We must, however, explicitly acknowledge the limitations inherent in a purely transcriptomic study. The presence and level of an mRNA transcript do not always directly correlate with the presence and functional activity of the corresponding protein, especially given the potential for maternally stored proteins, as we hypothesize for the cholinergic system. Therefore, these results should be viewed as a strong foundation for future proteomic and pharmacological studies required to confirm the functional activity of these systems.

## 5. Conclusions

In summary, our comparative transcriptomic analysis supports the concept that multiple neurotransmitter systems are transcriptionally represented and thus potentially active from the earliest stages of sea urchin development. These systems likely act simultaneously or sequentially, influencing both intracellular and intercellular signaling cascades in potentially synergistic or antagonistic ways. The remarkable diversity of neurotransmitter systems found in all adult tissues appears to be preprogrammed in the single egg cell, where they begin to function in a unique developmental context. Determining which of these numerous transcripts of transmitter components are converted into functionally competent proteins during oogenesis and early embryogenesis represents a key challenge for future research [1]. Further studies of the specific roles of these systems, their possible specialization in particular developmental processes, and the intricate interactions between the different transmitter pathways are needed to fully understand their collective contribution to the orchestration of early development.

## Figures and Tables

**Table 1:** Sampling stages of transcriptomes.

	EC	LC	EB	LB	EG
*M. franciscanus*	Egg	7 hpf	ND	16 hpf	29 hpf
*P. lividus*	Egg	ND	12 hpf	18 hpf	24 hpf
*L. variegatus*	1 hpf	2.5 hpf	4 hpf	7 hpf	12 hpf
*S. purpuratus*	Egg	10 hpf	18 hpf	24 hpf	30 hpf

EC—early cleavage; LC—late cleavage (about 60 blastomeres); EB—early blastula; LB—late blastula; EG—early gastrula; hpf—hours post fertilization; ND—no data.

**Table 2:** Values representing geometric mean of housekeeping genes expression (GHG) that were used to normalize transcriptome data.

Species	EC	LC	EB	LB	EG
*M. franciscanus*	559.068	2711.161	ND	1443.028	2004.563
*P. lividus*	97.394	ND	108.306	91.15	99.959
*L. variegatus*	4056.580	5140.630	4184.16	4166.32	7714.20
*S. purpuratus*	1388.124	5121.778	4838.051	6287.357	7629.709

EC—early cleavage; LC—late cleavage (about 60 blastomeres); EB—early blastula; LB—late (hatching) blastula; EG—early gastrula; ND—no data.

## Data Availability

The original contributions presented in this study are included in the article. Further inquiries can be directed to the corresponding author.

## Supplementary Materials

The following supporting information can be downloaded at: https://www.mdpi.com/article/10.3390/biology14091262/s1, Table S1: Expression of components of serotonergic mechanism; Table S2: Expression of components of dopaminergic mechanism; Table S3: Expression of components of adrenergic mechanism; Table S4: Expression of components of cholinergic mechanism; Table S5: Expression of components of GABA-ergic mechanism; Table S6: Expression of components of histaminergic mechanism; Table S7: Expression of components of glutamatergic mechanism; Figure S1: Single-cell transcriptomic analysis of three housekeeping genes chosen for normalization.

## Abbreviations

The following abbreviations are used in this manuscript:

5-HT	Serotonin
AADC	Aromatic L-amino acid decarboxylase
ACh	Acetylcholine
AChE	Acetylcholinesterase
adrR	Adrenergic receptor
AMPA	α-amino-3-hydroxy-5-methyl-4-isoxazolepropionic acid
ChAT	Choline-O-acetyltransferase
COMT	Catechol-O-methyltransferase
DAO	Diamine oxidase
DAT	Dopamine transporter
DBH	Dopamine-β-hydroxylase
EB	Early blastula
EC	Early cleavage
EG	Early gastrula
FPKM	Fragments Per Kilobase of transcript per Million mapped reads
GABA	Gamma-aminobutyric acid
GAD	Glutamate decarboxylase
GAPDH	Glyceraldehyde-3-phosphate dehydrogenase
GHG	Geometric mean of housekeeping genes
HDC	Histidine decarboxylase
HKG	Housekeeping genes
HNMT	Histamine N-methyltransferase
HPLC	High-performance liquid chromatography
HPRT	Hypoxanthine-guanine phosphoribosyltransferase
LB	Late blastula
LC	Late cleavage
AChR	Acetylcholine receptor
MAO	Monoamine oxidase
MZT	Maternal-to-zygotic transition
NET	Norepinephrine transporter
NMDA	N-methyl-D-aspartate
NO	Nitric oxide
NRPM	Normalized Reads Per Million
ODC	Ornithine decarboxylase
PAH	Phenylalanine hydroxylase
PCR	Polymerase chain reaction
RNA-Seq	RNA Sequencing
RPM	Reads Per Million
RT-PCR	Reverse transcription polymerase chain reaction
SERT	Serotonin transporter
TH	Tyrosine hydroxylase
TPM	Transcripts Per Million
TPH	Tryptophan 5-hydroxylase
TSA	Transcriptome Shotgun Assembly
VAChT	Vesicular acetylcholine transporter
VMAT2	Vesicular monoamine transporter 2
