# Supplementary material for: Expression Dynamics of Neurotransmitter System Genes in Early Sea Urchin Embryos: Insights from a Four-Species Comparative Transcriptome Analysis
Source: Biology (Basel). 2025 Sep 12;14(9):1262. doi: 10.3390/biology14091262 (PMC12467107; doi:10.3390/biology14091262)
Supplement: Supplementary file 1 [file biology-14-01262-s001.zip › Figure S1.pdf]

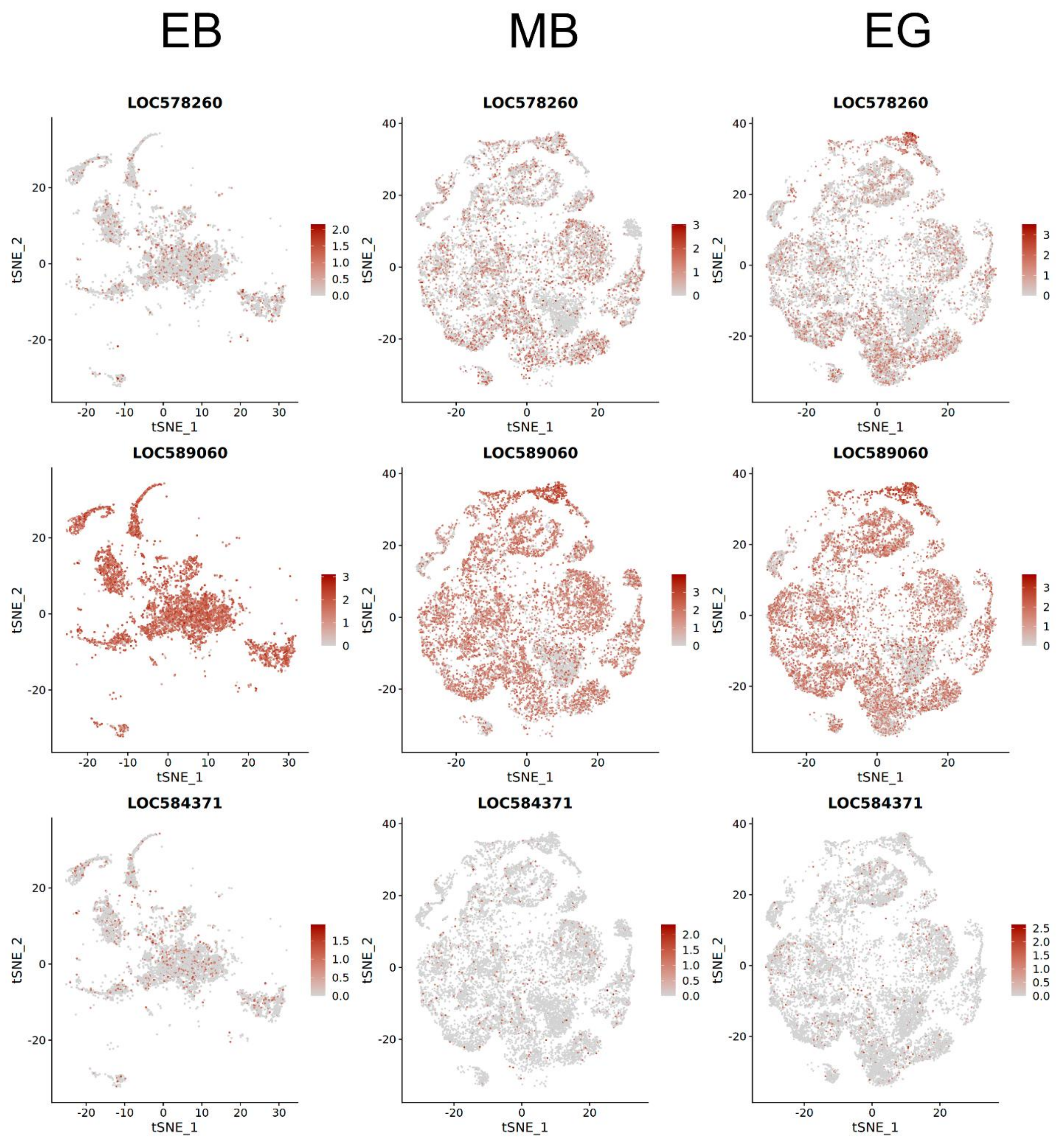

Figure S1. Single-cell transcriptomic analysis of the three housekeeping genes chosen for normalization, based on *S. purpuratus* data from [51]. Feature plots display the expression of *GAPDH* (LOC578260), *ODC* (LOC589060), and *HPRT* (LOC584371) at early blastula (EB), late (mesenchyme) blastula (MB), and early gastrula (EG) stages.
