# Supplementary material for: Expression Dynamics of Neurotransmitter System Genes in Early Sea Urchin Embryos: Insights from a Four-Species Comparative Transcriptome Analysis
Source: Biology (Basel). 2025 Sep 12;14(9):1262. doi: 10.3390/biology14091262 (PMC12467107; doi:10.3390/biology14091262)
Supplement: Supplementary file 1 [file biology-14-01262-s001.zip › S1.pdf]

Supplemental Table 1

Expression of the components of serotonergic mechanism

	Genes		Dev. Stages				
		M.fr	EC	LC		LB	EG
		S.pur	EC	LC	EB	LB	EG
		L.var	EC	LC	EB	LB	EG
		P.liv	EC		EB	LB	EG
Enzymes	*TPH*	*M.fr*	NS	NS		NS	NS
		S.pur	NS	0,222	0,061	0,421	0,074
		L.var	0,045	0,054	0,076	2,2	1,201
	AADC	*M.fr*	0,29	0,021		0,102	0,113
		S.pur	0,109	0,044	0,032	0,088	0,116
		L.var	1,813	1,813	1,368	0,51	0,355
		P.liv	0,004		1,783	0,195	0,372
	MAOA	*M.fr*	34,228	3,85		5,69	1,232
		S.pur	23,136	0,003	0,741	0,631	0,568
		L.var	4,38	2,959	1,761	0,13	0,06
		P.liv	12,947		0,127	0,275	0,082
Receptors	*HTR1A*	*M.fr*	0,32	0,181		0,116	0,195
		S.pur	0,032	NS	0,032	0,063	0,035
		L.var	0	NS	NS	0,003	NS
		P.liv	0,55		0,843	0,315	0,168
	HTR1B	*M.fr*	NS	NS		0,004	NS
		S.pur	0,427	0,072	0,007	0,007	0,01
		L.var	0,704	0,336	0,411	0,1	0,034
		P.liv	0,725		0,064	0,041	NS
	HTR1D	*M.fr*	0,58	0,175		0,15	0,174
	HTR1F	*M.fr*	0,39	0,224		0,403	0,183
	HTR6	*M.fr*	1,107	0,16		0,031	0,066
		S.pur	0,63	0,074	0,013	0,022	0,028
		L.var	0,777	0,551	0,587	0,034	0,039
		P.liv	0,18		0,043	0,09	0,141
Transporters	*SERT*	*S.pur*	0,022	0,006	NS	NS	NS
		L.var	0,019	0,013	NS	NS	NS
		P.liv	NS		0,009	0,005	NS
	VMAT	*S.pur*	NS	NS	NS	NS	0,005
		L.var	0,025	0,03	0,031	0,013	0,006
		P.liv	NS		0,007	0,008	0,01

NRPM (GHG)

Color bar:
≥ 5
4,0
3,0
2,0
1,0
0,5
0,4
0,3
0,2
0,1
0,01
0,003
0

Developmental Stages: EC - early cleavage; LC - late cleavage; EB - early blastula; LB - late blastula; EG - early gastrula. **Species names**: *M.fr - Mesocentrotus franciscanus; S.pur - Strongylocentrotus purpuratus; L.var - Lytechinus variegatus; P.liv - Paracentrotus lividus* . **Gene names**: *TPH* - tryptophan hydroxylase; *AADC* - aromatic L-amino acid decarboxylase; *HTR* - seotonin receptor; *SERT* - sodium-dependent serotonin transporter; *VMAT* - vesicular monoamine transporter. **Data definitions:** NRPM - RPM normalized to the geometric mean of the three housekeeping genes (GHG); NS - NS - not significant value. Transcriptomic data for this analysis were obtained from publicly available datasets:

1) Wong, J.M.; Gaitán-Espitia, J.D.; Hofmann, G.E. Transcriptional Profiles of Early Stage Red Sea Urchins (Mesocentrotus Franciscanus) Reveal Differential Regulation of Gene Expression across Development. Mar Genomics 2019, 48, 100692, doi:10.1016/j.margen.2019.05.007.

2) Hogan, J.D.; Keenan, J.L.; Luo, L.; Ibn-Salem, J.; Lamba, A.; Schatzberg, D.; Piacentino, M.L.; Zuch, D.T.; Core, A.B.; Blumberg, C.; et al. The Developmental Transcriptome for Lytechinus Variegatus Exhibits Temporally Punctuated Gene Expression Changes. Dev Biol 2020, 460, 139–154, doi:10.1016/j.ydbio.2019.12.002.

3) Gildor, T.; Malik, A.; Sher, N.; Avraham, L.; Ben-Tabou de-Leon, S. Quantitative Developmental Transcrip-tomes of the Mediterranean Sea Urchin Paracentrotus Lividus. Mar Genomics 2016, 25, 89–94, doi:10.1016/j.margen.2015.11.013.

4) Tu, Q.; Cameron, R.A.; Davidson, E.H. Quantitative Developmental Transcriptomes of the Sea Urchin Strongylocentrotus Purpuratus. Dev Biol 2014, 385, 160–167, doi:10.1016/j.ydbio.2013.11.019.
