# Supplementary material for: Expression Dynamics of Neurotransmitter System Genes in Early Sea Urchin Embryos: Insights from a Four-Species Comparative Transcriptome Analysis
Source: Biology (Basel). 2025 Sep 12;14(9):1262. doi: 10.3390/biology14091262 (PMC12467107; doi:10.3390/biology14091262)
Supplement: Supplementary file 1 [file biology-14-01262-s001.zip › S2.pdf]

Supplemental Table 2

Expression of the components of dopaminergic mechanism

	Genes		Dev. Stages				
	Genes	*M.fr*	EC	LC		LB	EG
		S.pur	EC	LC	EB	LB	EG
		L.var	EC	LC	EB	LB	EG
		P.liv	EC		EB	LB	EG
Enzymes	*TH*	*M.fr*	No data				
		S.pur	No data				
		L.var	No data				
		P.liv	No data				
	PAH	*S.pur*	0,021	0,214	1,439	0,547	0,372
		L.var	0,011	0,01	0,015	0,477	0,293
		P.liv	0,388		0,609	2,358	1,984
	MAOB	*S.pur*	NS	NS	0,005	NS	NS
		P.liv	0,24		0,207	0,113	0,082
	COMT	*M.fr*	0,064	0,006		0,014	0,01
		S.pur	0,025	0,0396	0,044	0,007	NS
		L.var	0,567	0,614	0,447	0,52	0,288
		P.liv	0,13		0,37	0,405	0,454
Receptors	*D1*	*M.fr*	1,15	0,344		0,151	0,252
		S.pur	0,404	0,042	0,016	0,034	0,055
		L.var	2,511	1,949	1,789	1,25	0,368
		P.liv	1,8		0,146	0,53	0,554
	D2	*M.fr*	0,23	0,059		0,061	0,066
		S.pur	0,004	NS	NS	NS	NS
		L.var	0,583	0,492	0,322	0,007	NS
		P.liv	0,034		0,013	0,006	NS
	D3	*M.fr*	NS	NS		0,008	NS
	D4	*S.pur*	0,006	NS	0,005	NS	NS
		L.var	0,004	0	0,009	0,027	NS
		P.liv	0,025		0,02	0,006	NS
	D5	*M.fr*	NS	NS		NS	NS
Transporter	*DAT*	*M.fr*	0,005	NS		0,075	0,237
		S.pur	0,035	0,012	NS	NS	NS
		L.var	0,011	0,01	0,006	0,013	0,004
		P.liv	0,014		NS	NS	NS

NRPM (GHG)

Color bar:
≥ 5
4,0
3,0
2,0
1,0
0,5
0,4
0,3
0,2
0,1
0,01
0,003
0

Developmental Stages: EC - early cleavage; LC - late cleavage; EB - early blastula; LB - late blastula; EG - early gastrula. **Species names**: *M.fr - Mesocentrotus franciscanus; S.pur - Strongylocentrotus purpuratus; L.var - Lytechinus variegatus; P.liv - Paracentrotus lividus* . **Gene names**: *TH* - tyrosine hydroxylase; *PAH* - phenylalanine hydroxylase; *MAOB* - monoamine oxidase B; *COMT* - catechol-O-methyltransferase; *DAT* - dopamine transporter. **Data definitions:** NRPM - RPM normalized to the geometric mean of the three housekeeping genes (GHG); NS - NS - not significant value. Transcriptomic data for this analysis were obtained from publicly available datasets:

1) Wong, J.M.; Gaitán-Espitia, J.D.; Hofmann, G.E. Transcriptional Profiles of Early Stage Red Sea Urchins (Mesocentrotus Franciscanus) Reveal Differential Regulation of Gene Expression across Development. Mar Genomics 2019, 48, 100692, doi:10.1016/j.margen.2019.05.007.

2) Hogan, J.D.; Keenan, J.L.; Luo, L.; Ibn-Salem, J.; Lamba, A.; Schatzberg, D.; Piacentino, M.L.; Zuch, D.T.; Core, A.B.; Blumberg, C.; et al. The Developmental Transcriptome for Lytechinus Variegatus Exhibits Temporally Punctuated Gene Expression Changes. Dev Biol 2020, 460, 139–154, doi:10.1016/j.ydbio.2019.12.002.

3) Gildor, T.; Malik, A.; Sher, N.; Avraham, L.; Ben-Tabou de-Leon, S. Quantitative Developmental Transcrip-tomes of the Mediterranean Sea Urchin Paracentrotus Lividus. Mar Genomics 2016, 25, 89–94, doi:10.1016/j.margen.2015.11.013.

4) Tu, Q.; Cameron, R.A.; Davidson, E.H. Quantitative Developmental Transcriptomes of the Sea Urchin Strongylocentrotus Purpuratus. Dev Biol 2014, 385, 160–167, doi:10.1016/j.ydbio.2013.11.019.
