# Supplementary material for: Expression Dynamics of Neurotransmitter System Genes in Early Sea Urchin Embryos: Insights from a Four-Species Comparative Transcriptome Analysis
Source: Biology (Basel). 2025 Sep 12;14(9):1262. doi: 10.3390/biology14091262 (PMC12467107; doi:10.3390/biology14091262)
Supplement: Supplementary file 1 [file biology-14-01262-s001.zip › S3.pdf]

Supplemental Table 3

Expression of the components of adrenergic mechanism

	Genes		Developmental Stages				
		M.fr	EC	LC		LB	EG
		S.pur	EC	LC	EB	LB	EG
		L.var	EC	LC	EB	LB	EG
		P.liv	EC		EB	LB	EG
Enzyme	*DBH*	*M.fr*	NS	0,003		0,005	NS
		S.pur	NS	NS	NS	NS	NS
		L.var	0,005	0,006	NS	0,008	0,023
		P.liv	0,019		0,02	0,017	0,007
α-Adrenoreceptors	*α1A-adrR*	*M.fr*	0,264	0,049		0,08	0,092
		S.pur	0,535	0,015	0,01	0,02	0,017
		L.var	1,013	0,994	1,009	1,02	1
		P.liv	NS		NS	NS	NS
	α1B-adrR	*M.fr*	NS	NS		0,004	NS
		L.var	0,016	0,043	0,006	0,353	NS
	α1D-adrR	*M.fr*	0,012	0,028		0,189	0,026
		L.var	0,742	0,662	1,84	0	18
	α2A-adrR	*L.var*	0,01	0,02	0,015	0,255	0
	α2B-adrR	*M.fr*	0,011	NS		NS	0,12
		L.var	0,016	0,007	0,006	5,138	0,333
	α2C-adrR	*M.fr*	0,026	0,023	0,02	0,027	0,004
β-Adrenoreceptors	*β1-adrR*	*M.fr*	0,053	0,014		0,043	0,133
		S.pur	0,016	0,07	0,054	NS	0,005
		L.var	0,073	0,069	0,0345	0,667	0,583
	β2-adrR	*M.fr*	0,036	0,04		0,23	0,052
		S.pur	4,969	0,511	0,095	0,082	0,079
		L.var	0,3027	0,225	0,2487	0,2	1,382
		P.liv	1,089		0,064	0,045	0,014
	β3-adrR	*M.fr*	0,024	0,01		0,009	NS
		S.pur	0,004	0,53	0,636	0,5	0,244
		L.var	0,067	0,03	0,029	1,553	0,972
		P.liv	0,899		0,597	0,394	0,495
Octopamine receptor	*OctR*	*M.fr*	0,067	0,007		0,012	NS
Tyramine receptor	*TyrR1*	*M.fr*	0,124	0,029		NS	NS
		L.var	0,028	0,026	0,031	0,128	0,079
Transporter	*NET*	*M.fr*	NS	NS		0,004	0,005
		S.pur	0,035	0,012	NS	NS	NS
		L.var	1,205	1,126	0,89	1,785	1,5
		P.liv	0,053		NS	NS	0,008

NRPM (GHG)

	Color bar:
≥	5
	4,0
	3,0
	2,0
	1,0
	0,5
	0,4
	0,3
	0,2
	0,1
	0,01
	0,003
	0

Developmental Stages: EC - early cleavage; LC - late cleavage; EB - early blastula; LB - late blastula; EG - early gastrula. **Species names**: *M.fr - Mesocentrotus franciscanus; S.pur - Strongylocentrotus purpuratus; L.var - Lytechinus variegatus; P.liv - Paracentrotus lividus* . **Gene names**: *DBH* - Dopamine-β-hydroxylase; *AdrR* – adrenoreceptor; *OctR* - octopamine receptor; *TyrR* - tyramine receptor; *NET* - noadrenaline ransporter. **Data definitions:** NRPM - RPM normalized to the geometric mean of the three housekeeping genes (GHG); NS - NS - not significant value.

Transcriptomic data for this analysis were obtained from publicly available datasets:

1) Wong, J.M.; Gaitán-Espitia, J.D.; Hofmann, G.E. Transcriptional Profiles of Early Stage Red Sea Urchins (Mesocentrotus Franciscanus) Reveal Differential Regulation of Gene Expression across Development. Mar Genomics 2019, 48, 100692, doi:10.1016/j.margen.2019.05.007.

2) Hogan, J.D.; Keenan, J.L.; Luo, L.; Ibn-Salem, J.; Lamba, A.; Schatzberg, D.; Piacentino, M.L.; Zuch, D.T.; Core, A.B.; Blumberg, C.; et al. The Developmental Transcriptome for Lytechinus Variegatus Exhibits Temporally Punctuated Gene Expression Changes. Dev Biol 2020, 460, 139–154, doi:10.1016/j.ydbio.2019.12.002.

3) Gildor, T.; Malik, A.; Sher, N.; Avraham, L.; Ben-Tabou de-Leon, S. Quantitative Developmental Transcrip-tomes of the Mediterranean Sea Urchin Paracentrotus Lividus. Mar Genomics 2016, 25, 89–94, doi:10.1016/j.margen.2015.11.013.

4) Tu, Q.; Cameron, R.A.; Davidson, E.H. Quantitative Developmental Transcriptomes of the Sea Urchin Strongylocentrotus Purpuratus. Dev Biol 2014, 385, 160–167, doi:10.1016/j.ydbio.2013.11.019.
