# Supplementary material for: Expression Dynamics of Neurotransmitter System Genes in Early Sea Urchin Embryos: Insights from a Four-Species Comparative Transcriptome Analysis
Source: Biology (Basel). 2025 Sep 12;14(9):1262. doi: 10.3390/biology14091262 (PMC12467107; doi:10.3390/biology14091262)
Supplement: Supplementary file 1 [file biology-14-01262-s001.zip › S4.pdf]

Supplemental Table 4

Expression of the components of cholinergic mechanism

	Genes		Dev. Stages				
		M.fr	EC	LC		LB	EG
		S.pur	EC	LC	EB	LB	EG
		L.var	EC	LC	EB	LB	EG
		P.liv	EC		EB	LB	EG
Enzymes	*ChAT*	*M.fr*	No data				
		S.pur	No data				
		L.var	No data				
		P.liv	No data				
	AChE	*M.fr*	0,613	0,077		0,094	0,013
		S.pur	0,128	0,06	0,036	0,103	0,082
		L.var	0,528	0,381	0,359	0,097	0,032
		P.liv	NS		NS	NS	NS
	ChE1	*M.fr*	2,701	0,827		2,481	3,429
	ChE2	*M.fr*	0,139	0,024		0,027	0,069
Muscarinic receptors	*M1-AChR*	*M.fr*	NS	NS		NS	NS
		L.var	0,045	0,041	0,019	0,028	NS
	M2-AChR	*M.fr*	0,097	0,048		0,064	0,121
		S.pur	0,74	0,577	1,388	0,033	0,2
		P.liv	0,422		0,03	0,032	0,041
	M3-AChR	*M.fr*	0,013	0,014		0,012	NS
		S.pur	0,015	NS	NS	0,006	NS
		L.var	0,378	0,322	1,128	0,043	0,004
	M4-AChR	*M.fr*	0,021	0,018		0,009	0,009
		L.var	NS	NS	NS	NS	NS
	M5-AChR	*M.fr*	0,165	0,2638		0,0062	NS
		S.pur	NS	0,007	0,045	0,012	0,014
		L.var	0,213	0,142	0,021	0,072	0,011
		P.liv	0,035		0,011	0,004	NS
Nicotinic receptor subunits	*nAChR α1*	*L.var*	0,006	0,008	0,031	0,095	0,047
	nAChR α2	*M.fr*	38,08	3,974		11,221	6,359
		L.var	0,02	0,011	0,017	0,019	0,019
		P.liv	NS		NS	NS	NS
	nAChR α3	*M.fr*	5,203	0,328		0,768	0,63
	nAChR α5	*L.var*	NS	NS	NS	NS	0,019
	nAChR α6	*S.pur*	0,086	0,018	0,007	0,01	0,087
		L.var	1,111	1,079	1,09	1,136	1,149
		P.liv	0,004		0,02	0,139	0,123
	nAChR α7	*M.fr*	0,035	NS		0,008	NS
		S.pur	0,356	0,774	1,583	0,096	0,067
		L.var	5,54	2,76	3,337	0,153	0,061
		P.liv	2,087		1,112	0,713	0,658
	nAChR α8	*S.pur*	0,02	0,009	NS	0,01	NS
	nAChR α9	*M.fr*	NS	0,004		0,007	0,003
		S.pur	0,078	0,008	0,004	NS	0,003
		L.var	0,126	0,069	0,1	0,05	0,009
		P.liv	0,118		0,064	0,012	0,014
	nAChR α10	*M.fr*	1,167	0,254		0,385	0,343
		L.var	0,123	0,091	NS	0,003	0,02
	nAChR β3	*M.fr*	2,929	0,375		0,184	0,815
		S.pur	NS	0,006	0,003	0,004	NS
		P.liv	0,054		0,03	0,004	NS
Transporter	*VAChT*	*M.fr*	0,005	NS		NS	0,008
		L.var	0,009	0,011	0,025	0,004	0,006
		P.liv	NS		NS	NS	NS

NRPM (GHG)

Color bar:
≥ 5
4,0
3,0
2,0
1,0
0,5
0,4
0,3
0,2
0,1
0,01
0,003
0

Developmental Stages: EC - early cleavage; LC - late cleavage; EB - early blastula; LB - late blastula; EG - early gastrula. **Species names**: *M.fr - Mesocentrotus franciscanus; S.pur - Strongylocentrotus purpuratus; L.var - Lytechinus variegatus; P.liv - Paracentrotus lividus* . **Gene names:** *ChAT* - choline-O-acetyltransferase; *AChE* - acetylcholine esterase; *ChE* - choline esterase; *mAChR* - muscarinic cholinoreceptor; *nAChR* - nicotinic cholinoreceptor; *VAChT* - vesicular acetylcholine transporter. **Data definitions:** NRPM - RPM normalized to the geometric mean of the three housekeeping genes (GHG); NS - NS - not significant value. Transcriptomic data for this analysis were obtained from publicly available datasets:

1) Wong, J.M.; Gaitán-Espitia, J.D.; Hofmann, G.E. Transcriptional Profiles of Early Stage Red Sea Urchins (Mesocentrotus Franciscanus) Reveal Differential Regulation of Gene Expression across Development. Mar Genomics 2019, 48, 100692, doi:10.1016/j.margen.2019.05.007.

2) Hogan, J.D.; Keenan, J.L.; Luo, L.; Ibn-Salem, J.; Lamba, A.; Schatzberg, D.; Piacentino, M.L.; Zuch, D.T.; Core, A.B.; Blumberg, C.; et al. The Developmental Transcriptome for Lytechinus Variegatus Exhibits Temporally Punctuated Gene Expression Changes. Dev Biol 2020, 460, 139–154, doi:10.1016/j.ydbio.2019.12.002.

3) Gildor, T.; Malik, A.; Sher, N.; Avraham, L.; Ben-Tabou de-Leon, S. Quantitative Developmental Transcrip-tomes of the Mediterranean Sea Urchin Paracentrotus Lividus. Mar Genomics 2016, 25, 89–94, doi:10.1016/j.margen.2015.11.013.

4) Tu, Q.; Cameron, R.A.; Davidson, E.H. Quantitative Developmental Transcriptomes of the Sea Urchin Strongylocentrotus Purpuratus. Dev Biol 2014, 385, 160–167, doi:10.1016/j.ydbio.2013.11.019.
