# Supplementary material for: Expression Dynamics of Neurotransmitter System Genes in Early Sea Urchin Embryos: Insights from a Four-Species Comparative Transcriptome Analysis
Source: Biology (Basel). 2025 Sep 12;14(9):1262. doi: 10.3390/biology14091262 (PMC12467107; doi:10.3390/biology14091262)
Supplement: Supplementary file 1 [file biology-14-01262-s001.zip › S5.pdf]

Supplemental Table 5

Expression of the components of GABA-ergic mechanism

<table>
<tr><td colspan="3"></td><td colspan="6">Dev. Stages</td></tr>
<tr><td colspan="2" rowspan="4">Genes</td><td>M.fr</td><td>EC</td><td colspan="2">LC</td><td colspan="2">LB</td><td>EG</td></tr>
<tr><td>S.pur</td><td>EC</td><td>LC</td><td colspan="2">EB</td><td>LB</td><td>EG</td></tr>
<tr><td>L.var</td><td>EC</td><td>LC</td><td colspan="2">EB</td><td>LB</td><td>EG</td></tr>
<tr><td>P.liv</td><td colspan="2">EC</td><td colspan="2">EB</td><td>LB</td><td>EG</td></tr>
<tr><td rowspan="3">Enzyme</td><td rowspan="3">GAD</td><td>M.fr</td><td>0,421</td><td colspan="2">0,129</td><td colspan="2">0,217</td><td>0,758</td></tr>
<tr><td>S.pur</td><td>0,000</td><td>0,000</td><td colspan="2">0,039</td><td>0,023</td><td>0,007</td></tr>
<tr><td>L.var</td><td>NS</td><td>NS</td><td colspan="2">NS</td><td>NS</td><td>NS</td></tr>
<tr><td rowspan="9">Receptors</td><td rowspan="3">GABA A1</td><td>S.pur</td><td>0,014</td><td>0,006</td><td colspan="2">0,007</td><td>NS</td><td>NS</td></tr>
<tr><td>L.var</td><td>NS</td><td>NS</td><td colspan="2">NS</td><td>NS</td><td>0,004</td></tr>
<tr><td>P.liv</td><td colspan="2">NS</td><td colspan="2">NS</td><td>NS</td><td>NS</td></tr>
<tr><td rowspan="3">GABA B1</td><td>M.fr</td><td>1,778</td><td colspan="2">0,548</td><td colspan="2">0,081</td><td>0,036</td></tr>
<tr><td>S.pur</td><td>0,437</td><td>0,387</td><td colspan="2">0,021</td><td>NS</td><td>NS</td></tr>
<tr><td>P.liv</td><td colspan="2">NS</td><td colspan="2">NS</td><td>NS</td><td>NS</td></tr>
<tr><td rowspan="3">GABA B2</td><td>M.fr</td><td>0,829</td><td colspan="2">0,135</td><td colspan="2">0,032</td><td>NS</td></tr>
<tr><td>S.pur</td><td>0,036</td><td>0,033</td><td colspan="2">0,012</td><td>0,005</td><td>0,003</td></tr>
<tr><td>P.liv</td><td colspan="2">NS</td><td colspan="2">NS</td><td>NS</td><td>NS</td></tr>
<tr><td rowspan="4">Transporter</td><td rowspan="4">GABAT</td><td>M.fr</td><td>1,15</td><td colspan="2">0,446</td><td colspan="2">0,167</td><td>0,213</td></tr>
<tr><td>S.pur</td><td>NS</td><td>NS</td><td colspan="2">NS</td><td>NS</td><td>NS</td></tr>
<tr><td>L.var</td><td>0,452</td><td>0,405</td><td colspan="2">0,397</td><td>0,023</td><td>0,033</td></tr>
<tr><td>P.liv</td><td colspan="2">0,014</td><td colspan="2">0,013</td><td>0,007</td><td>NS</td></tr>
</table>

NRPM (GHG)

Color bar:

≥	5
	4,0
	3,0
	2,0
	1,0
	0,5
	0,4
	0,3
	0,2
	0,1
	0,01
	0,003
	0

Developmental Stages: EC - early cleavage; LC - late cleavage; EB - early blastula; LB - late blastula; EG - early gastrula. **Species names**: *M.fr - Mesocentrotus franciscanus; S.pur - Strongylocentrotus purpuratus; L.var - Lytechinus variegatus; P.liv - Paracentrotus lividus* . **Gene names**: *GAD* - glutamate decarboxylase; *GABAT* - GABA-transporter. **Data definitions:** NRPM - RPM normalized to the geometric mean of the three housekeeping genes (GHG); NS - NS - not significant value. Transcriptomic data for this analysis were obtained from publicly available datasets:

1) Wong, J.M.; Gaitán-Espitia, J.D.; Hofmann, G.E. Transcriptional Profiles of Early Stage Red Sea Urchins (Mesocentrotus Franciscanus) Reveal Differential Regulation of Gene Expression across Development. Mar Genomics 2019, 48, 100692, doi:10.1016/j.margen.2019.05.007.

2) Hogan, J.D.; Keenan, J.L.; Luo, L.; Ibn-Salem, J.; Lamba, A.; Schatzberg, D.; Piacentino, M.L.; Zuch, D.T.; Core, A.B.; Blumberg, C.; et al. The Developmental Transcriptome for Lytechinus Variegatus Exhibits Temporally Punctuated Gene Expression Changes. Dev Biol 2020, 460, 139–154, doi:10.1016/j.ydbio.2019.12.002.

3) Gildor, T.; Malik, A.; Sher, N.; Avraham, L.; Ben-Tabou de-Leon, S. Quantitative Developmental Transcrip-tomes of the Mediterranean Sea Urchin Paracentrotus Lividus. Mar Genomics 2016, 25, 89–94, doi:10.1016/j.margen.2015.11.013.

4) Tu, Q.; Cameron, R.A.; Davidson, E.H. Quantitative Developmental Transcriptomes of the Sea Urchin Strongylocentrotus Purpuratus. Dev Biol 2014, 385, 160–167, doi:10.1016/j.ydbio.2013.11.019.
