# Supplementary material for: Expression Dynamics of Neurotransmitter System Genes in Early Sea Urchin Embryos: Insights from a Four-Species Comparative Transcriptome Analysis
Source: Biology (Basel). 2025 Sep 12;14(9):1262. doi: 10.3390/biology14091262 (PMC12467107; doi:10.3390/biology14091262)
Supplement: Supplementary file 1 [file biology-14-01262-s001.zip › S6.pdf]

Supplemental Table 6

Expression of the components of histaminergic mechanism

			Dev. Stages				
	Genes	*M.fr*	EC	LC		LB	EG
		S.pur	EC	LC	EB	LB	EG
		L.var	EC	LC	EB	LB	EG
		P.liv	EC		EB	LB	EG
Enzymes	*HDC*	*M.fr*	NS	NS		NS	0,034
		S.pur	1,781	0,222	0,253	0,307	0,192
		P.liv	NS		NS	NS	NS
	HNMT	*M.fr*	NS	NS		NS	0,034
		S.pur	0,934	0,499	0,811	0,165	0,071
		L.var	0,017	0,008	0,013	0,01	NS
		P.liv	NS		NS	NS	0,004
	DAO	*S.pur*	0,147	0,154	0,096	0,188	0,074
		L.var	0,056	0,062	0,064	0,045	0,029
		P.liv	0,021		0,005	0,009	0,036
Receptors	*H1*	*M.fr*	0,013	0,039		0,077	0,035
		S.pur	0,045	0,007	NS	0,005	0,006
		L.var	0,632	0,649	1,054	0,154	0,067
	H2	*M.fr*	0,377	0,328		0,021	0,022
		S.pur	0,821	0,016	0,025	0,036	0,046
		L.var	0,906	0,79	1,623	0,245	0,135
	H3	*M.fr*	0,049	0,005		0,012	NS
		S.pur	0,483	0,016	0,037	NS	0,023
		L.var	0,09	0,083	0,11	0,004	0,009
	H4	*M.fr*	NS	NS		NS	NS
		S.pur	0,004	NS	NS	NS	NS
		L.var	0,007	0,006	0,004	NS	0,005
		P.liv	NS		NS	NS	NS

NRPM (GHG)

Color bar:

≥	5
	4,0
	3,0
	2,0
	1,0
	0,5
	0,4
	0,3
	0,2
	0,1
	0,01
	0,003
	0

Developmental Stages: EC - early cleavage; LC - late cleavage; EB - early blastula; LB - late blastula; EG - early gastrula. **Species names**: *M.fr - Mesocentrotus franciscanus; S.pur - Strongylocentrotus purpuratus; L.var - Lytechinus variegatus; P.liv - Paracentrotus lividus* . **Gene names**: *HDC* - histidine decarboxylase; *HNMT* - histamine N-methyltransferase; *DAO* - diamine oxidase. **Data definitions:** NRPM - RPM normalized to the geometric mean of the three housekeeping genes (GHG); NS - NS - not significant value. Transcriptomic data for this analysis were obtained from publicly available datasets:

1) Wong, J.M.; Gaitán-Espitia, J.D.; Hofmann, G.E. Transcriptional Profiles of Early Stage Red Sea Urchins (Mesocentrotus Franciscanus) Reveal Differential Regulation of Gene Expression across Development. Mar Genomics 2019, 48, 100692, doi:10.1016/j.margen.2019.05.007.

2) Hogan, J.D.; Keenan, J.L.; Luo, L.; Ibn-Salem, J.; Lamba, A.; Schatzberg, D.; Piacentino, M.L.; Zuch, D.T.; Core, A.B.; Blumberg, C.; et al. The Developmental Transcriptome for Lytechinus Variegatus Exhibits Temporally Punctuated Gene Expression Changes. Dev Biol 2020, 460, 139–154, doi:10.1016/j.ydbio.2019.12.002.

3) Gildor, T.; Malik, A.; Sher, N.; Avraham, L.; Ben-Tabou de-Leon, S. Quantitative Developmental Transcrip-tomes of the Mediterranean Sea Urchin Paracentrotus Lividus. Mar Genomics 2016, 25, 89–94, doi:10.1016/j.margen.2015.11.013.

4) Tu, Q.; Cameron, R.A.; Davidson, E.H. Quantitative Developmental Transcriptomes of the Sea Urchin Strongylocentrotus Purpuratus. Dev Biol 2014, 385, 160–167, doi:10.1016/j.ydbio.2013.11.019.
