# Supplementary material for: Expression Dynamics of Neurotransmitter System Genes in Early Sea Urchin Embryos: Insights from a Four-Species Comparative Transcriptome Analysis
Source: Biology (Basel). 2025 Sep 12;14(9):1262. doi: 10.3390/biology14091262 (PMC12467107; doi:10.3390/biology14091262)
Supplement: Supplementary file 1 [file biology-14-01262-s001.zip › S7.pdf]

Supplemental Table 7

Expression of the components of glutamatergic mechanism

	Genes		Dev. Stages				
		M.fr	EC	LC		LB	EG
		S.pur	EC	LC	EB	LB	EG
		L.var	EC	LC	EB	LB	EG
		P.liv	EC		EB	LB	EG
Metabotropic receptors	*Grm1*	*M.fr*	8,856	0,843		4,519	1,593
	Grm2	*M.fr*	NS	NS		NS	0,016
	Grm3	*M.fr*	30,339	3,968		6,929	1,264
		S.pur	0,173	0,048	0,025	0,004	0,017
		L.var	3,454	3,706	6,534	0,22	0,073
		P.liv	0,761		0,034	0,052	0,073
	Grm3-2	*M.fr*	2,136	0,66		7,011	14,804
	Grm4	*S.pur*	0,229	0,029	0,045	0,03	0,033
	Grm6	*S.pur*	NS	NS	NS	NS	NS
		L.var	0,864	0,78	0,904	0,402	0,282
		P.liv	0,016		0,005	0,007	0,018
	Grm7	*M.fr*	0,074	0,053		0,01	0,058
	Grm8	*M.fr*	0,056	0,015		NS	NS
		S.pur	0,133	0,013	0,028	0,023	0,029
		L.var	0,77	0,601	0,819	0,249	0,113
Ionotropic receptors	*Gria1*	*S.pur*	16,857	2,196	7,353	4,763	5,834
		L.var	2,644	2,577	2,196	6,925	5,352
		P.liv	1,023		1,814	12,538	27,54
	Glur2	*S.pur*	16,138	2,496	1,621	1,638	0,306
	Glur3	*S.pur*	10,484	1,401	3,103	2,955	0,345
	Grik1 (kainate)	*M.fr*	NS	NS		NS	NS
		S.pur	0,065	0,014	0,004	NS	NS
		P.liv	0,315		0,055	0,056	0,045
Transporter	*GluT*	*M.fr*	0,302	0,107		0,171	0,193

NRPM (GHG)

Color bar:	
≥	5
	4,0
	3,0
	2,0
	1,0
	0,5
	0,4
	0,3
	0,2
	0,1
	0,01
	0,003
	0

Developmental Stages: EC - early cleavage; LC - late cleavage; EB - early blastula; LB - late blastula; EG - early gastrula. **Species names**: *M.fr - Mesocentrotus franciscanus; S.pur - Strongylocentrotus purpuratus; L.var - Lytechinus variegatus; P.liv - Paracentrotus lividus* . **Gene names**: *Grm* - metabotropic glutamate receptor; *Gria, Glur* - ionotropic AMPA-receptors; *GluT* - glutamate transporter. **Data definitions**: NRPM - RPM normalized to the geometric mean of the three housekeeping genes (GHG); NS - NS - not significant value. Transcriptomic data for this analysis were obtained from publicly available datasets:

1) Wong, J.M.; Gaitán-Espitia, J.D.; Hofmann, G.E. Transcriptional Profiles of Early Stage Red Sea Urchins (Mesocentrotus Franciscanus) Reveal Differential Regulation of Gene Expression across Development. Mar Genomics 2019, 48, 100692, doi:10.1016/j.margen.2019.05.007.

2) Hogan, J.D.; Keenan, J.L.; Luo, L.; Ibn-Salem, J.; Lamba, A.; Schatzberg, D.; Piacentino, M.L.; Zuch, D.T.; Core, A.B.; Blumberg, C.; et al. The Developmental Transcriptome for Lytechinus Variegatus Exhibits Temporally Punctuated Gene Expression Changes. Dev Biol 2020, 460, 139–154, doi:10.1016/j.ydbio.2019.12.002.

3) Gildor, T.; Malik, A.; Sher, N.; Avraham, L.; Ben-Tabou de-Leon, S. Quantitative Developmental Transcrip-tomes of the Mediterranean Sea Urchin Paracentrotus Lividus. Mar Genomics 2016, 25, 89–94, doi:10.1016/j.margen.2015.11.013.

4) Tu, Q.; Cameron, R.A.; Davidson, E.H. Quantitative Developmental Transcriptomes of the Sea Urchin Strongylocentrotus Purpuratus. Dev Biol 2014, 385, 160–167, doi:10.1016/j.ydbio.2013.11.019.
